# Plastome comparison and phylogenomics of *Fagopyrum* (Polygonaceae): insights into sequence differences between *Fagopyrum* and its related taxa

**DOI:** 10.1186/s12870-022-03715-5

**Published:** 2022-07-14

**Authors:** Qiu-jie Li, Yu Liu, An-hu Wang, Qing-fu Chen, Jian-mei Wang, Lu Peng, Yi Yang

**Affiliations:** 1 College of Life Sciences, Sichuan University, Chengdu, 610065 China; 2 Panxi Crops Research and Utilization Key Laboratory of Sichuan Province, Xichang University, Xichang, 615013 China; 3 Research Center of Buckwheat Industry Technology, Guizhou Normal University, Guiyang, 550001 China

**Keywords:** *Fagopyrum*, Plastome variation, Phylogenomics, Polygonaceae, Inverted repeat boundary, Genome size, Species relationship

## Abstract

**Background:**

*Fagopyrum* (Polygonaceae) is a small plant lineage comprised of more than fifteen economically and medicinally important species. However, the phylogenetic relationships of the genus are not well explored, and the characteristics of *Fagopyrum* chloroplast genomes (plastomes) remain poorly understood so far. It restricts the comprehension of species diversity in *Fagopyrum*. Therefore, a comparative plastome analysis and comprehensive phylogenomic analyses are required to reveal the taxonomic relationship among species of *Fagopyrum*.

**Results:**

In the current study, 12 plastomes were sequenced and assembled from eight species and two varieties of *Fagopyrum*. In the comparative analysis and phylogenetic analysis, eight previously published plastomes of *Fagopyrum* were also included. A total of 49 plastomes of other genera in Polygonaceae were retrieved from GenBank and used for comparative analysis with *Fagopyrum*. The variation of the *Fagopyrum* plastomes is mainly reflected in the size and boundaries of inverted repeat/single copy (IR/SC) regions. *Fagopyrum* is a relatively basal taxon in the phylogenomic framework of Polygonaceae comprising a relatively smaller plastome size (158,768–159,985 bp) than another genus of Polygonaceae (158,851–170,232 bp). A few genera of Polygonaceae have nested distribution of the IR/SC boundary variations. Although most species of *Fagopyrum* show the same IRb/SC boundary with species of Polygonaceae, only a few species show different IRa/SC boundaries. The phylogenomic analyses of *Fagopyrum* supported the cymosum and urophyllum groups and resolved the systematic position of subclades within the urophyllum group. Moreover, the repeat sequence types and numbers were found different between groups of *Fagopyrum*. The plastome sequence identity showed significant differences between intra-group and inter-group.

**Conclusions:**

The deletions of intergenic regions cause a short length of *Fagopyrum* plastomes, which may be the main reason for plastome size diversity in Polygonaceae species. The phylogenomic reconstruction combined with the characteristics comparison of plastomes supports grouping within *Fagopyrum*. The outcome of these genome resources may facilitate the taxonomy, germplasm resources identification as well as plant breeding of *Fagopyrum*.

**Supplementary Information:**

The online version contains supplementary material available at 10.1186/s12870-022-03715-5.

## Background

**Fig. 1:**
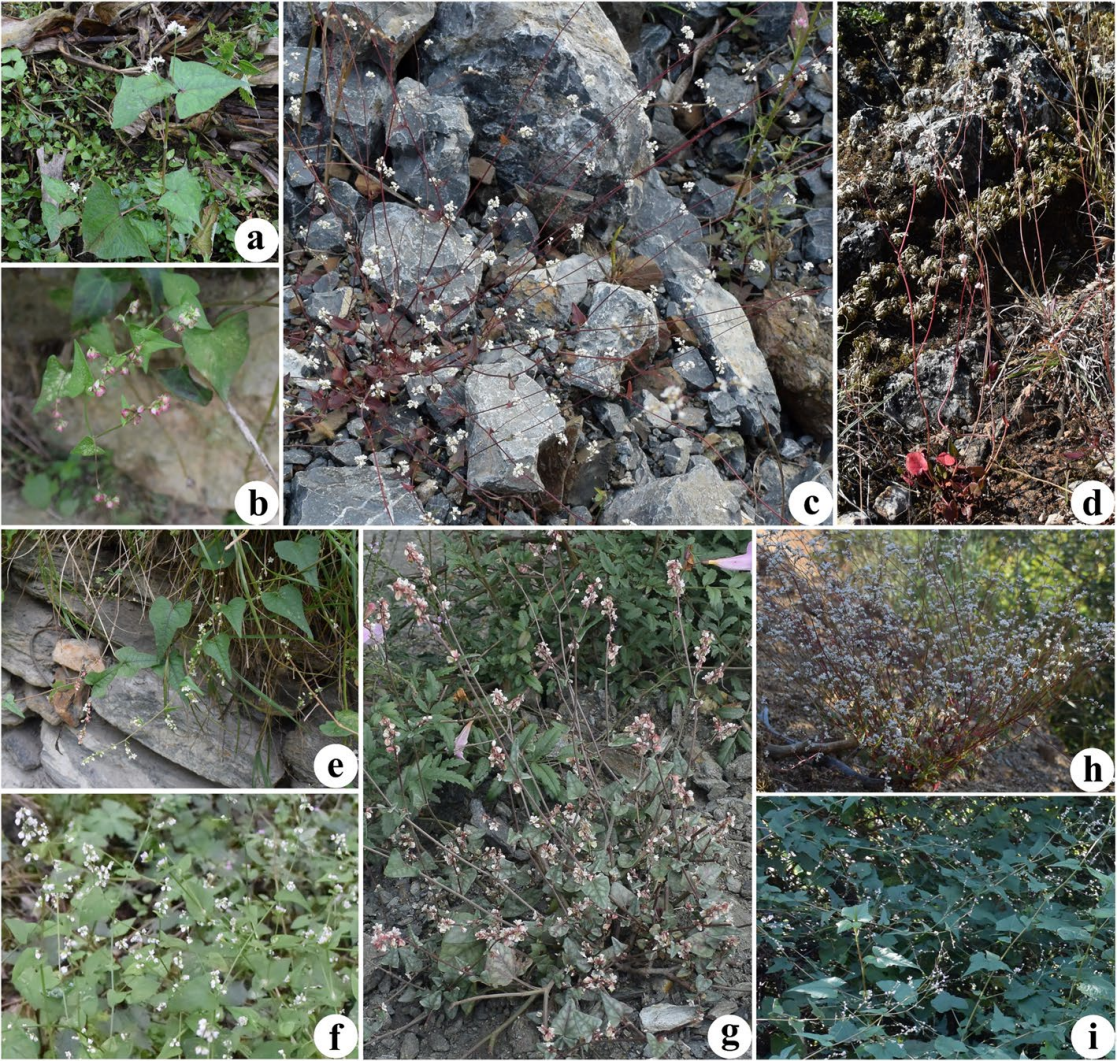
Field photographs of *Fagopyrum* taxa show divergent morphological features. **a** *F. cymosum* (Photo was taken from Jiguanshan, Chongzhou, Sichuan, China); **b** *F. gracilipes* var. *odontopterum* (Photo was taken from Yangchang, Dafang, Guizhou, China); **c** *F. leptopodum* var. *grossii* (Photo was taken from Jinsha, Leibo, Sichuan, China); **d** *F. statice* (Photo was taken from Yumeidu, Shilin, Yunnan, China); **e** *F. caudatum* (Photo was taken from Tonghua, Wenchuan, Sichuan, China);** f** *F. gracilipes* (Photo was taken from Waziping, Dujiangyan, Sichuan, China); **g** *F. qiangcai* (Photo was taken from Bashinao, Lixian, Sichuan, China); **h** *F. leptopodum* (Photo was taken from Ala, Panzhihua, Sichuan, China);** i** *F. urophyllum* (Photo was taken from Jizushan, Binchuan, Yunnan, China)

The genus *Fagopyrum* belongs to the family Polygonaceae and includes approximately 15 to 28 species [1–5]. Most of the wild species of *Fagopyrum* are narrowly distributed in mountainous areas of southwest China. Several endemic species are only distributed to the southeastern edge of the Qinghai-Tibetan Plateau [2, 4], and this area is considered as the birthplace of the two cultivated buckwheat species, namely *Fagopyrum esculentum* and *F. tataricum* [6]. The known cultivated species *F. esculentum*, is a cereal that produce gluten-free grains [7]. Additionally, the seeds of Tartary buckwheat (*F. tataricum*) are used as important functional food ingredients [8], and the rhizome of *F. cymosum* (*F. dibotrys*) is used as a pharmaceutical drug in lung diseases treatment [9]. The genus *Fagopyrum* has morphological diversity with annual and perennial plants, including herbs, woody lianas, and shrubs (Fig. 1). Plants in the genus *Fagopyrum* have attracted the attention of botanists because of the economic importance of their cultivars and the potential use of their wild genetic resources [10, 11].

The assessment of the phylogenetic relationships among *Fagopyrum* species is a prerequisite of an efficient breeding program, as it provides a basis for selecting desirable genotypes [12]. In the classical taxonomy based on the inflorescence, coverage of the achene with the perianth, and achene size, the genus *Fagopyrum* is divided into two major taxa, i.e., *F. esculentum *sensu lato and *F. gilesii *sensu lato [13, 14]. In phylogenetic studies based on DNA sequences of ITS, *rbcL*, and *accD*, *Fagopyrum* is classified into two groups, i.e., cymosum group and urophyllum group, which agree with the two taxa described in earlier taxonomic studies [12, 15, 16]. The cymosum group corresponds to *F. esculentum *sensu lato and includes the species *F. cymosum*, *F. esculentum*, and *F. tataricum*. The urophyllum group corresponds to *F. gilesii *sensu lato and includes the species *F. gilesii*, *F. gracilipes*, *F. leptopodum*, *F. statice*, and *F. urophyllum*. Recent studies have also supported this division in *Fagopyrum* [2, 4, 17]; for example, the species belonging to the big-achene group are distinguished from those in the small-achene group in terms of their persistent perianths, which are longer than the seeds, as well as their larger seeds [2]. Additionally, based on the DNA sequences of *psbE-psbL* and *ndhA* intron the *Fagopyrum* species were divided into the cultivated group and the wild group by the phylogenetic analyses [17]. Although these findings demonstrate the existence of two distinct groups in *Fagopyrum*, due to the limitation associated with plant materials or robust molecular data, the relationships among the species remain obscure within these two groups [4].

In recent years, new species of *Fagopyrum* have been reported based on morphological characteristics [5, 18–23]. *F. hailuogouense*, reported as a new species of *Fagopyrum*, was later shown to belong to the genus *Bistorta* using molecular evidence [24, 25]. *F. wenchuanense* was originally reported to be closely related to *F. gracilipes* and classified into the urophyllum group, but it was later categorized into the cymosum group based on molecular data [12, 22]. Therefore, molecular data are required to support the taxonomic status of these new species.

Chloroplast genomes (plastomes) are rich sources of phylogenetic data and are widely used in phylogenetic studies due to their moderate nucleotide substitution rates, uniparental inheritance, conserved structure, ease of sequencing, as well as great abundance of existing data [26–28]. The molecular markers such as highly divergent regions and repeat sequences derived from the entire plastome sequence hold promise for use in species delimitation and population genetics [29–31]. Moreover, the comparison of complete plastome sequences further provides the opportunity to explore sequence variation and molecular evolutionary patterns associated with gene loss, rearrangements, duplication, and transfer events [32–34]. Until now, plastomes have only been used for comparative analyses of single species of *Fagopyrum* or a few species of the genus [35, 36]. Compared with phylogenetic studies limited to a few complete plastomes or a few plastid loci, plastome phylogenomic studies provide much greater resolution [37–39]. Thus, a comprehensive plastome analysis in *Fagopyrum* is much needed at the infrageneric level.

In contrast to previous studies, we sampled a taxonomically representative set of species within the cymosum and the urophyllum group in *Fagopyrum*. To characterize plastomes, comparative analyses were used 20 plastomes of *Fagopyrum* and 49 other genus plastomes of Polygonaceae (Table S1, S2). The phylogenomic analyses were performed to explore systematic positions and relationships of species in *Fagopyrum*. The objectives of this study included (1) characterization of the plastome variation of *Fagopyrum*; (2) to test whether the plastome data could resolve current uncertainties in the phylogeny of the *Fagopyrum*; (3) to investigate the genetic diversity of *Fagopyrum* useful in the identification of wild germplasm resources and improvement of cultivated variety and breed.

## Results

### Characteristics of *Fagopyrum* plastomes

**Fig. 2:**
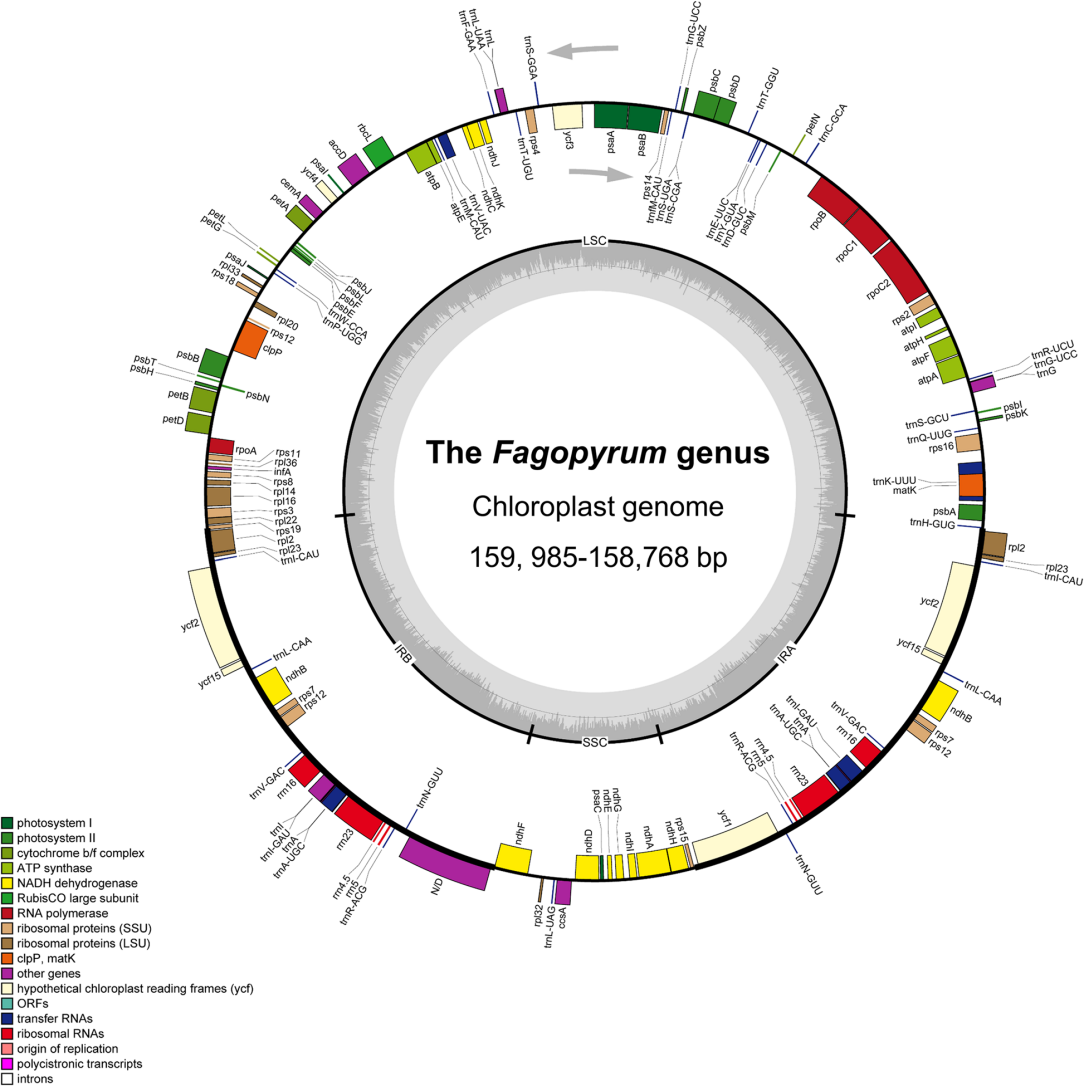
The plastome map of *Fagopyrum*. Genes shown outside the circle are transcribed clockwise, whereas genes shown inside the circle are transcribed counterclockwise. The color of genes differentiates the protein-coding genes based on their respective functions. The AT and GC contents of the genome are plotted on the inner circle as light and dark grey, respectively. The inverted repeats are denoted as IRa and IRb; the large single-copy is denoted as LSC, and the small single-copy regions are denoted as SSC

A total of 12 *Fagopyrum* plastomes were obtained in our study. The number of paired-end raw reads obtained by the Illumina sequencing ranged from 6, 778, 507 to 25, 542, 740. The number of reads mapped to *Fagopyrum* plastomes ranged from 446, 898 to 2, 356, 508 and the average coverage depth ranges from 426 × to 5265 × (Table 1). The 12 high-quality plastome sequences were deposited in the GenBank with accession numbers MZ491847, MZ702791–MZ702801 (Table S1). A representative plastome map of *Fagopyrum* was drawn using OGDRAW, as shown in Fig. 2. The GC contents of these 12 newly sequenced *Fagopyrum* plastomes ranged from 37.8% to 37.9%. A total of 131 genes were annotated, and 86 protein-coding genes (72 single-copy genes and seven genes with two copies), 37 tRNA genes (19 single-copy genes and nine genes with two copies), and eight rRNA genes (four genes with two copies) were identified (Table 2). In *Fagopyrum* species, gene numbers and orders were found to be conserved, similar to those of many Polygonaceae genera (Table S3). The multiple sequence alignments in *Fagopyrum* were performed using mVISTA software that revealed the high sequence similarity (> 90%) of 12 newly sequenced plastomes (Fig. S1).

**Table 1:** The quantity of the sequencing data and coverage depth of the 12 assembled plastomes

Species	Raw reads no	Mapped reads No	coverage ( ×)
*F. megaspartanium*	18, 203, 454	1, 917, 714	1810
*F. cymosum*	20, 895, 137	1, 375, 223	1291
*F. statice*	14, 703, 147	1, 655, 635	1551
*F. leptopodum var. grossii*	6, 778, 507	669, 765	1132
*F. leptopodum*	15, 258, 779	836, 663	818
*F. urophyllum* (lianas)	17, 566, 621	446, 898	426
*F. sp*	19, 824, 585	939, 432	906
*F. urophyllum*	25, 542, 740	1, 012, 882	969
*F. qiangcai*	15, 396, 608	2, 356, 508	2215
*F. caudatum*	13, 997, 722	167, 898	681
*F. gracilipes* var. *odontopterum*	12, 750, 680	5, 614, 317	5265
*F. gracilipes*	20, 659, 431	898, 566	2716

**Table 2:** Plastomes characteristics of newly sequenced *Fagopyrum* species

Taxon	Species	Genome (bp)	LSC (bp)	IR (bp)	SSC (bp)	Gene content	GC content(%)
cymosum group	*F. cymosum*	159,935	85,038	30,792	13,313	86/37/8	37.9
	*F. megaspartanium*	159,985	85,051	30,793	13,348	86/37/8	37.9
urophyllum group	*F. caudatum*	159,197	84,347	30,844	13,162	86/37/8	37.8
	*F. gracilipes* var. *odontopterum*	159,297	84,455	30,841	13,160	86/37/8	37.8
	*F. gracilipes*	159,097	84,209	30,848	13,192	86/37/8	37.9
	*F. leptopodum*	158,768	83,902	30,839	13,188	86/37/8	37.9
	*F. leptopodum var. grossii*	159,343	84,444	30,840	13,219	86/37/8	37.8
	*F. qiangcai*	159,144	84,531	30,729	13,155	86/37/8	37.8
	*F. sp*	159,341	84,449	30,848	13,196	86/37/8	37.9
	*F. statice*	159,265	84,392	30,841	13,191	86/37/8	37.8
	*F. urophyllum* (lianas)	159,427	84,520	30,846	13,215	86/37/8	37.8
	*F. urophyllum*	159,288	84,425	30,846	13,171	86/37/8	37.8

*LSC* Large single copy region, *SSC* Small single copy region, *IR* Inverted repeat region

### Plastome size

**Fig. 3:**
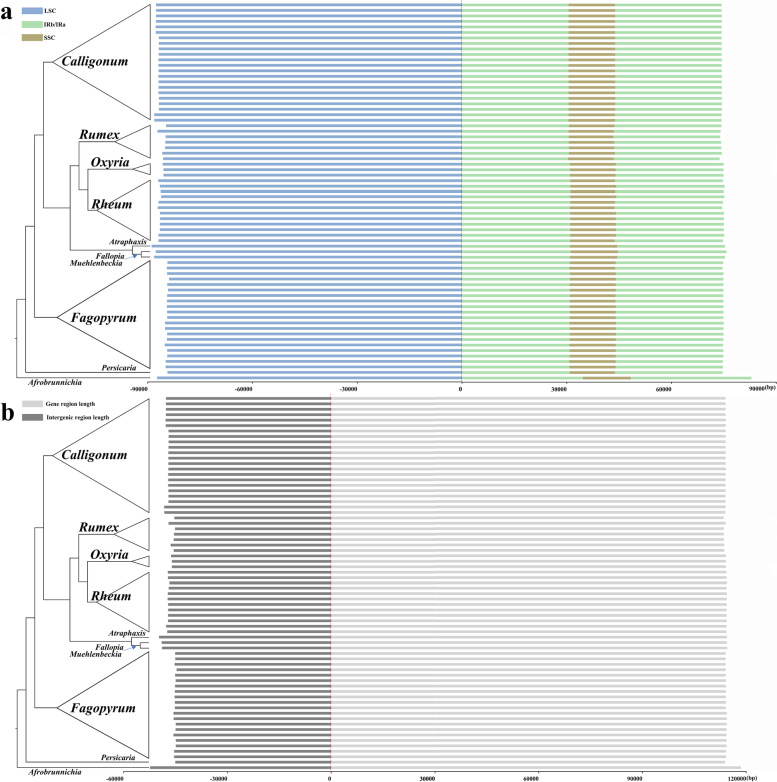
The relevance between the expansion/contraction of LSC-IRs and the plastome-based phylogeny.** a** Left side is the phylogenetic framework reconstructed using 69 complete plastome sequences. Right side is the length stacked bar chart of the four constituent regions (LSC, IRb, SSC, and IRa) of plastomes. The blue bars represent LSC regions, green bars represent IRs. (IRb and IRa), and brown bars represent SSC regions. **b** Right side is the length stacked bar chart of the gene and intergenic regions of plastomes. The black bars represent intergenic regions, and gray bars represent gene regions

This study summarizes the complete sequence length and lengths of the four constituent regions (LSC, IRb, SSC, and IRa) from 69 plastomes of Polygonaceae species. To analyze the correlation between expansion/contraction of IR/SC of the plastomes and phylogeny, a length stacked bar chart was constructed according to the phylogenetic framework (Fig. 3, Table S4). The *Fagopyrum* clade was located at the base of the phylogenomic framework of Polygonaceae. The complete sequence of plastomes in *Fagopyrum* ranges from 158,768 bp (*Fagopyrum leptopodum*) to 159,985 bp (*Fagopyrum megaspartanium*). The LSC region was found located between 83,902–85,135 bp, with the IR region and the SSC region spanning from 30,685–30,870 bp and 13,093–13,348 bp, respectively (Fig. 3a, Table S4). The gene region was found between 114, 081–114, 406 bp as well as an intergenic region was spanning between 44,678–45,598 bp (Fig. 3b, Table S4). In the Polygonaceae except for *Fagopyrum*, the species with the largest (170,974 bp) and smallest (158,981 bp) plastome sizes were *Afrobrunnichia erecta* and *Persicaria chinensis* respectively, both the species were located at the base of the phylogenomic framework. The length of LSC regions, IR region and SSC region are between 84,347–88,878 bp, 30,348–34,631 bp and 12,762–13,653 bp, respectively (Fig. 3a, Table S4). Furthermore, the length of gene region was between 113,671–117,858 bp, and the length of intergenic region was between 45,174–53,374 bp (Fig. 3b, Table S4).

### IR/SC boundaries

**Fig. 4:**
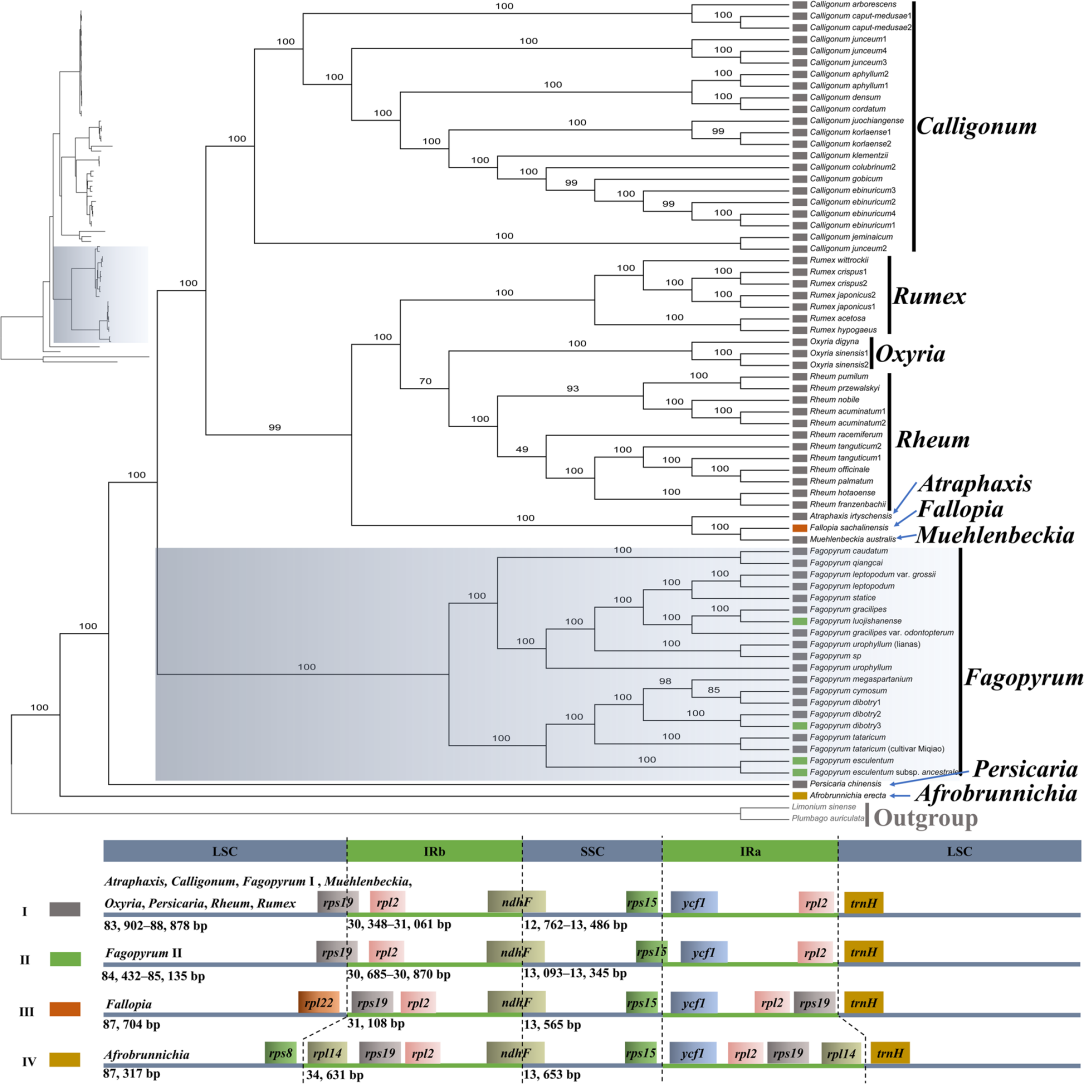
Phylogenetic relationships of the Polygonaceae, inferred by maximum likelihood (ML) based on complete plastome sequences. Accessions of the species used in the phylogenetic tree are provided in Table S1, S2. Values above branches are bootstrap support (BS)**.** Illustrations represent the junctions of the inverted repeat (IR) and the single-copy (SC) regions. The color blocks in the phylogenetic tree correspond to different types of IR/SC boundaries

The IR/SC boundaries among 20 *Fagopyrum* plastomes and 49 plastomes of other genera in Polygonaceae were compared. In Polygonaceae*,* four types of IR/SC boundaries were summarized and presented in the phylogenetic tree (Fig. 4). The type I IR/SC boundary was found most common among the 12 newly obtained plastomes of *Fagopyrum* and in most genera of Polygonaceae, such as *Calligonum*, *Muehlenbeckia*, *Oxyria*, *Persicaria*, *Rheum*, and some species of *Rumex*. In this type of boundary, the *rps19* and *ndhF* genes straddled the LSC/IRb and IRb/SSC boundary regions respectively. The SSC/IRa boundary genes were *rps15* and *ycf1*, while *rpl2* and *trnH* were the IRa/LSC boundary genes. The type II IR/SC boundary was found in some species of *Fagopyrum* (*F. esculentum*, *F. esculentum* subsp. *ancestrale*, *F. dibotry3,* and *F. luojishanense*), similar to the type I boundary, except that the *rps15* gene straddled the SSC/IRa boundary region. The type III IR/SC and type IV boundary were found in *Fallopia* and *Afrobrunnichia,* respectively. The type III and type IV IR/SC boundaries similar to the type I boundary, except that *rps19* and *rpl14* were present in the IRb region, and the IR region expanded to a greater degree in the type IV boundary compared with the type III boundary. A detailed comparison of the IR/SC boundaries has been presented in Fig S2, S3.

### Sequence diversity and hotspots

**Fig. 5:**
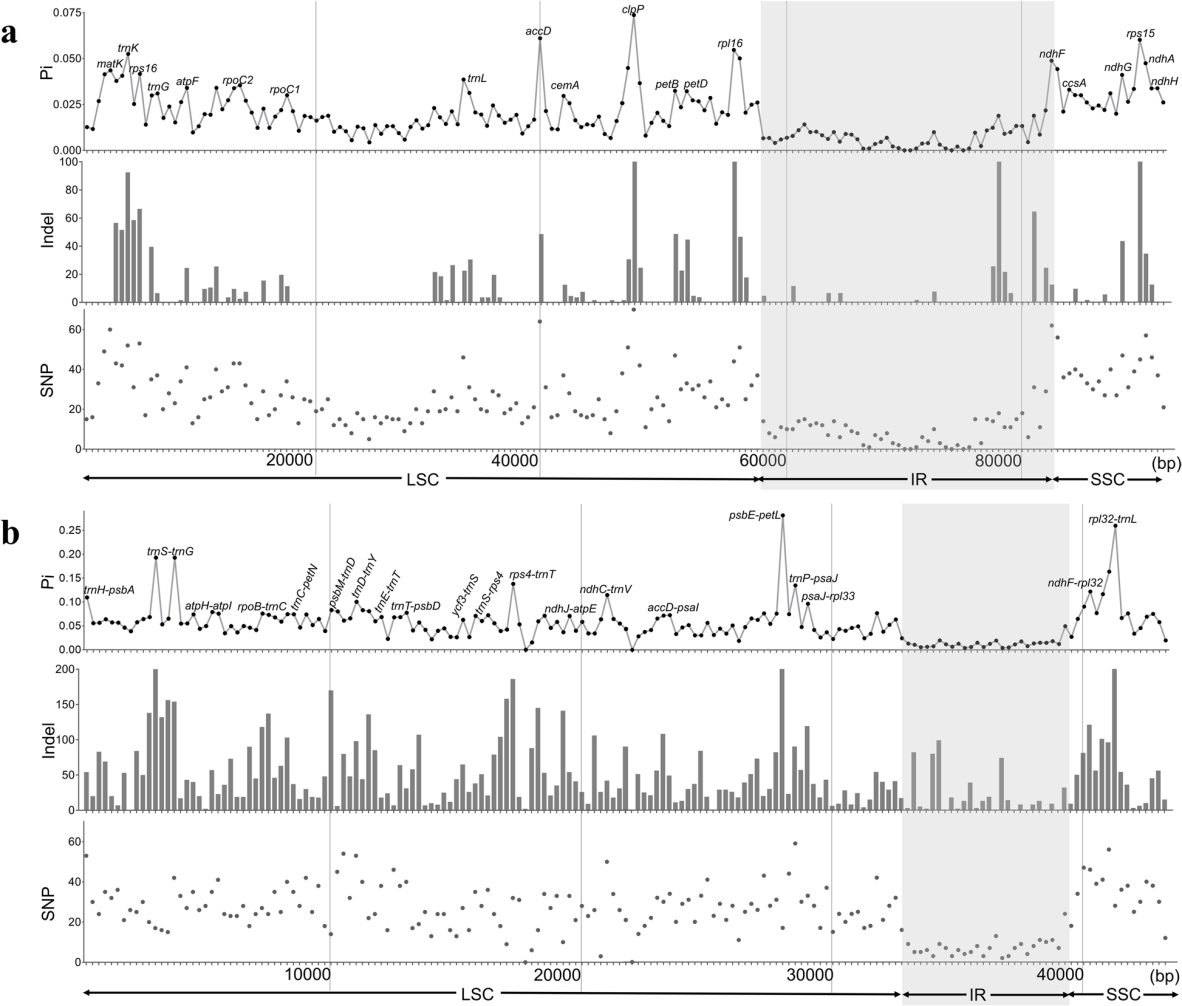
Phylogenetic informativeness (Pi), insertions/deletions (indels) and polymorphic sites (SNPs) among the plastomes of *Fagopyrum*. **a** Sequence diversity of gene regions. **b** Sequence diversity of intergenic regions. The X-axis shows the regions in plastomes, while the Y-axis represents the Pi, indels and SNPs at each region. The LSC, IR, and SSC on the X-axis indicate large single-copy, inverted repeat, and small single-copy regions, respectively. The gray shaded regions represent the inverted repeat (IR) regions

The single nucleotide polymorphisms (SNPs), insertions/deletions (indels), as well as phylogenetic informativeness (Pi) of the 184 non-overlapping matrices of gene region and 173 non-overlapping matrices of the intergenic region were calculated based on multiple plastome sequences in *Fagopyrum*. The gene matrix with the highest Pi value was found in *clpP* gene regions, followed by *accD* and *rps15* genes. On the other hand, the intergenic matrix with the highest Pi value was observed in *psbE-petL* gene region, followed by *rpl32-trnL* and *trnS-trnG* genes (Fig. 5). The genes or intergenic regions with high Pi value matrices were identified as hotspots. The Pi value of the top 20 hotspots of gene regions ranged from 0.02617 to 0.05215, whereas for the intergenic regions, it ranged from 0.05079 to 0.13248 (Table S6). The result of variation analysis showed that the LSC and SSC regions were more divergent than the two IR regions and had a higher divergence in the intergenic regions than gene regions. The top 20 hotspots of neither the gene regions nor the intergenic regions were found in the IR region (Fig. 5).

The Ka/Ks ratios of the 79 protein-coding genes are shown in Fig. S4, Table S5. The majority (96.20%) of Ka/Ks ratios was between 0 and 1. The average Ka/Ks ratio for cymosum group plastome genes was 0.1996, and for urophyllum group plastome genes was 0.1743 (Fig. S4a, b). There were 66 plastome genes with synonymous and non-synonymous substitutions in the cymosum group, but only 42 in the urophyllum group. In cymosum group, four gene (*rps15*, *petN*, *ycf2*, *ycf3*) had Ka/Ks rate higher than 1 (Fig. S4b). In urophyllum group, nine gene (*ndhD*, *rps12*, *rpoA*, *rpoC2*, *ycf1*, *ycf2*, *accD*, *ccsA*, *matK*) had Ka/Ks rate higher than 1 (Fig. S4a).

### Repeat sequences

**Fig. 6:**
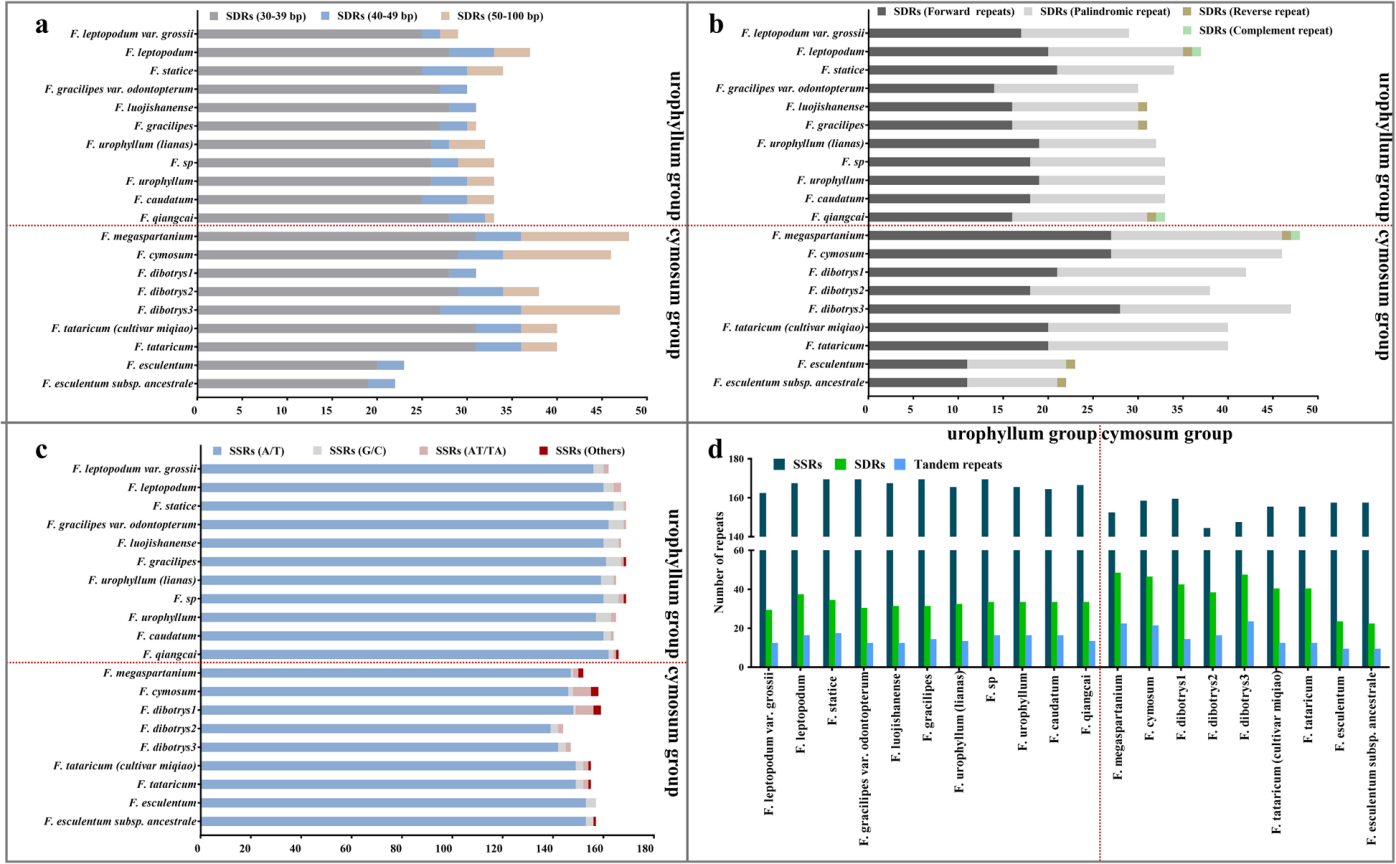
Comparison of repetitive sequences among *Fagopyrum* species. **a** Represent short dispersed repeats (SDRs) that present in specific range of size i.e., 30–39 represent numbers of repeats within the size range of 30 and 39 bp. **b** Describe number of different types of SDRs. **c** Represent repeats that present different types of simple sequence repeats (SSRs). **d** Represent numbers of different types repeats in 12 *Fagopyrum* species 50%

Short dispersed repeats (SDRs) (including forward repeats, reverse repeats, complement repeats, and palindromic repeats), simple sequence repeats (SSRs) as well as tandem repeats were analyzed in this study (Fig. 6). The results of plastome SDRs analysis showed considerable variations in the numbers and length of repeats in different *Fagopyrum* groups. The plastomes with the most numbers of SDRs (*F. megaspartanium*) and with the least number of SDRs (*F. esculentum* subsp. *ancestrale*) were found in the cymosum group (Fig. 6a). In four types of SDRs, the most found was forward repeats, followed by reverse repeats and complement repeats. The forward and reverse repeats exist in each plastome of *Fagopyrum*, the complement repeats exist in seven plastomes of *Fagopyrum*. The palindromic repeats are least found, exist only in plastomes of *F. qiangcai*, *F. leptopodum*, and *F. megaspartanium* (Fig. 6b).

The number of SSRs found in the plastomes of the cymosum group, and urophyllum group was 144–159 and 162–169, respectively. Most of the SSRs were mono-nucleotide repeats (98.32%) having the bases of mono-nucleotide repeat sequences adenine/thymine (97.63%) (Fig. 6c). The number of tandem repeats in plastomes for the cymosum group and urophyllum groups was 9–22 and 12–17, respectively. There were obvious differences between the cymosum and urophyllum groups in terms of the number of SSRs, SDRs, or tandem repeats (Fig. 6d).

### Plastome sequence identity

**Fig. 7:**
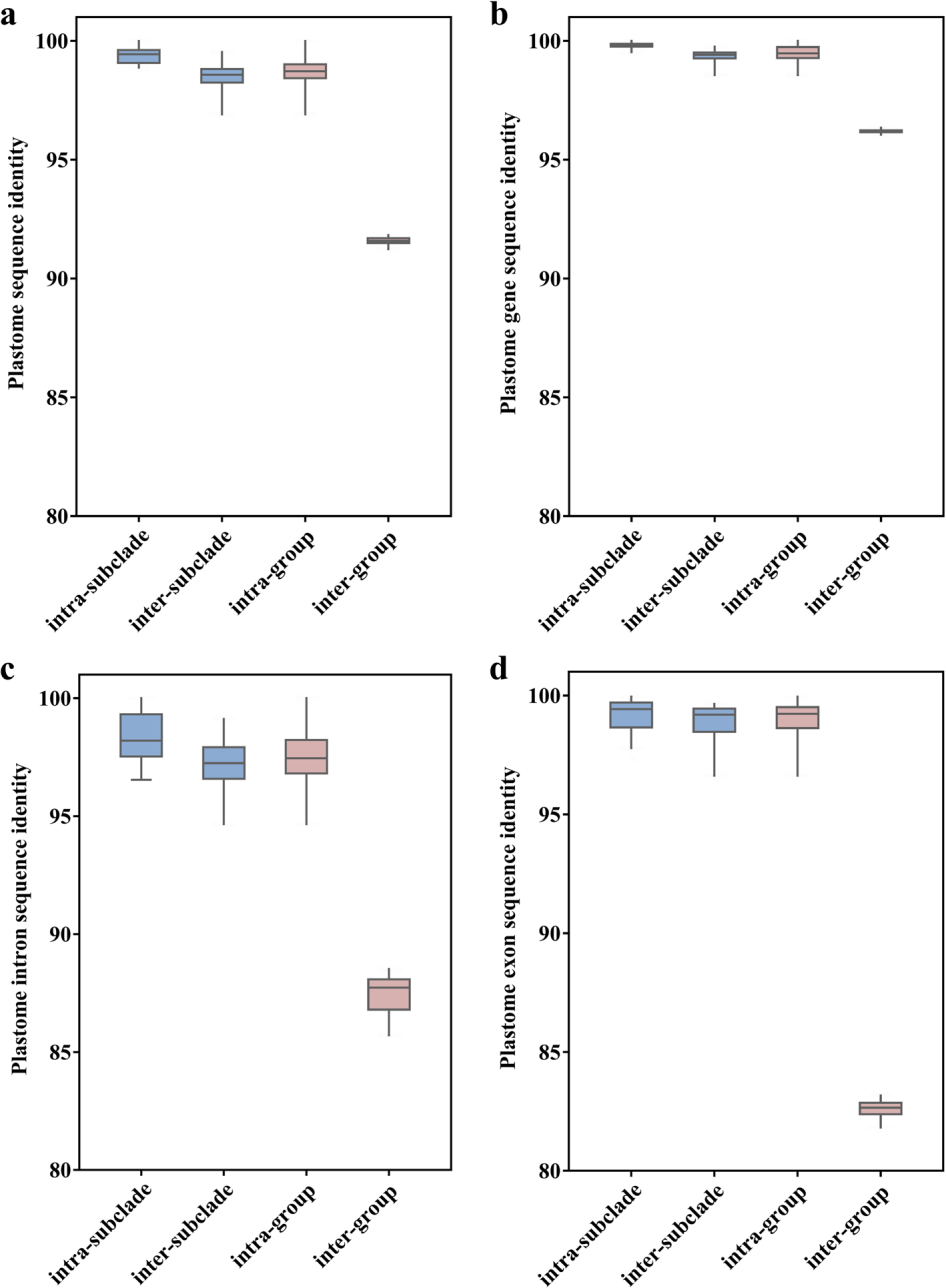
Plastome sequence identity of *Fagopyrum.* **a** The complete plastome sequence identify. **b** The plastome gene sequence identify. **c** The plastome intron sequence identify. **d** The plastome exon sequence identify. The pink squares represent sequence identity of species in same group or between different group. The blue squares represent sequence identity of species in same subclade or between different subclade but in same group

The comparison of plastome revealed the high sequence identity in groups among *Fagopyrum* (Fig. 7). The intra-group plastome sequence identity significantly higher than inter-group. Among the cymosum group, the sequence identity of plastome was found from 97.01% to 99.75%, whereas in the urophyllum group, it ranges from 98.22% to 99.69%. The sequence identity of plastome between cymosum and urophyllum group, was from 91.24% to 91.83% (Fig. 7a). The difference of sequence identity between intra-group and inter-group also occurs in plastome gene regions (Fig. 7b). The largest difference in sequence identity between intra-group and inter-group occurred in exon sequences, rather than intron sequences (Fig. 7c, d).

### Phylogenetic analysis

**Fig. 8:**
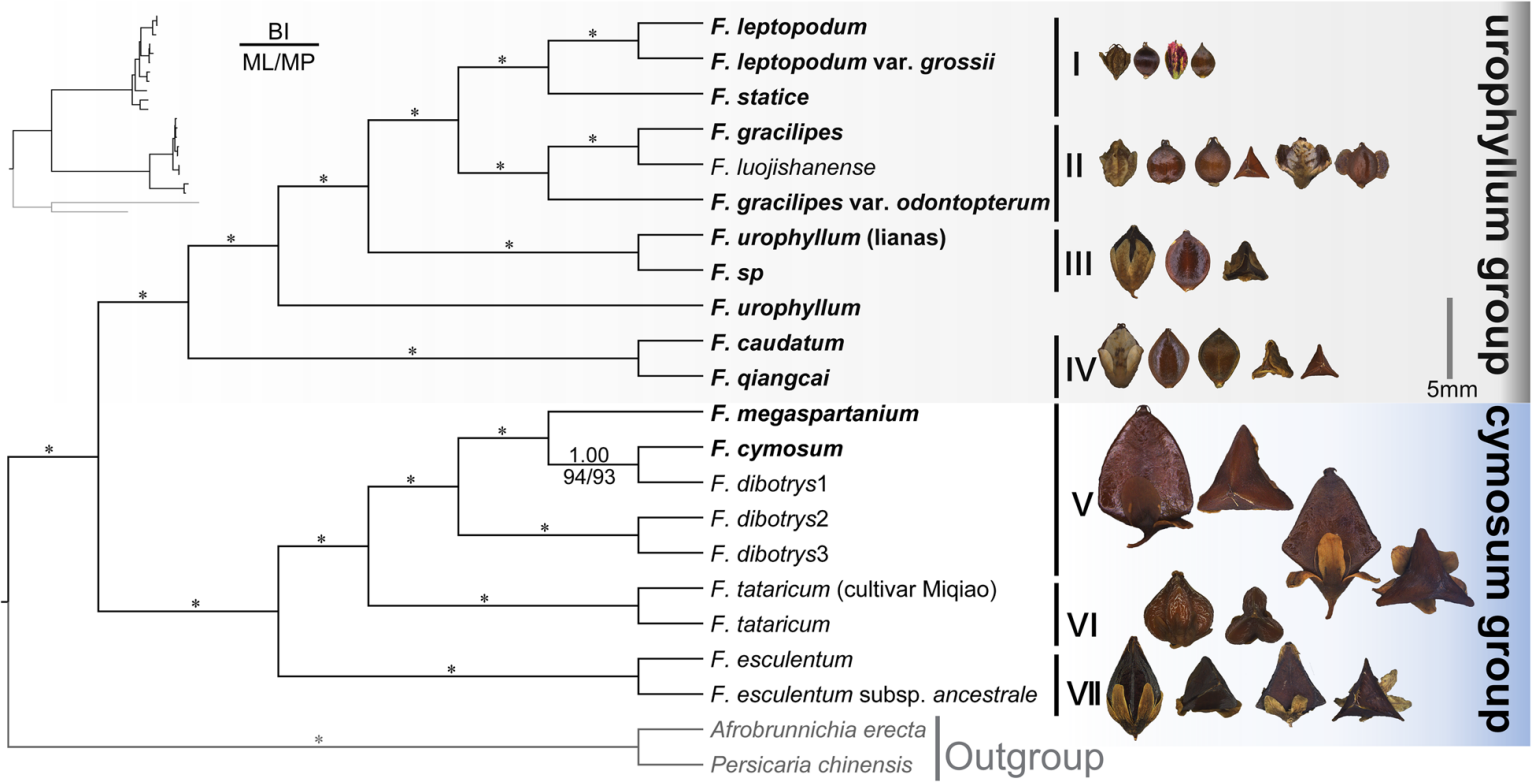
Phylogenetic relationships within the *Fagopyrum* based on the complete plastome sequences. The size of achenes is on the same scale. Values above branches are posterior probabilities (PP), and values below branches are bootstrap support (BS) percentages, inferred from maximum-likelihood analyses (left) and maximum parsimony (right), * represent a best support (1.00 or 100)

A total of 69 complete plastome sequences of Polygonaceae and two plastomes of outgroup were used for the phylogenomic inference (Table S2). Phylogenomic tree constructed by the ML method, provides strong support for the monophyly of *Fagopyrum* (bootstrap support (BS) = 100) (Fig. 4). The genus *Fagopyrum* clustered at the base of Polygonaceae as a sister clade with *Calligonum*, *Fallopia*, *Oxyria*, *Rheum*, and *Rumex* genera. In *Fagopyrum*, the phylogenomic relationships inferred from the BI, ML, and MP analyses were consistent (Fig. 8), and the tree showed the formation of two monophyletic clades, i.e., the cymosum group and the urophyllum group, with strong support [BS = 100, posterior probabilities (PP) = 1]. Seven subclades were recovered within the *Fagopyrum*, including(I) *F. leptopodum*, *F. leptopodum var. grossii*, and *F. statice*; (II) *F. gracilipes*, *F. gracilipes* var. *odontopterum*, and *F. luojishanense*; (III) *F. urophyllum* (lianas) and *F.* sp.; (IV) *F. caudatum* and *F. qiangcai*; (V) *F. megaspartanium*, *F. cymosum*, *F. dibotrys*1, *F. dibotrys*2, and *F. dibotrys*3; (VI) *F. tataricum* and *F. tataricum* (cultivar Miqiao); (VII) *F. esculentum* and *F. esculentum* subsp. *ancestrale*. The subclades I–IV belonged to the urophyllum group clade, while subclades V–VII belonged to the cymosum group clade.

To identify the useful molecular markers, six data matrices of *Fagopyrum* plastome sequences were used to construct phylogenies. The results demonstrated that the tree topologies generated based on matrices consisting of all gene regions, 20 gene regions, and eight gene regions were consistent with the tree, constructed based on the complete plastome sequences. The topological structure of the subclade of *F. cymosum* and *F. megaspartanium* showed a difference between the trees based on complete plastome sequences and matrices of all intergenic regions, 20 intergenic regions, and eight intergenic regions. Similarly, the topological structure of the subclade of *F. caudatum* and *F. qiangcai* and the subclade of *F. gracilipes* revealed the difference between the trees based on complete plastome sequences and matrices of eight intergenic regions (Fig. S5, Table S6).

## Discussion

### Plastome variation

The comparative analysis of plastome sequences provides new insights into sequence variation and molecular evolutionary patterns [27, 39]. The current study found conserved patterns among plastomes of *Fagopyrum* were conserved in the quadripartite structure, length, gene order, and GC content (Fig. S2, Table 2, S3). These results were found similar to previous studies that explored a few species of *Fagopyrum* [36, 40]. Different from previous studies, a detailed plastome characteristics comparison between *Fagopyrum* with other Polygonaceae genera was performed in this study. These results revealed the variations of the *Fagopyrum* plastomes that are mainly reflected in intergenic regions length and SSC/IRa boundary. It is important to note that the results of these plastome comparative analyses were presented in conjunction with the results of phylogenetic analyses in order to show the plastome variation more intuitively in different taxa. Such as combining plastome size with phylogenetic framework (Fig. 3) and mapping SC/IR boundary types to phylogenetic trees (Fig. 4). This approach of analysis, which links plastome sequence variation to phylogenetic position, has improved our understanding of molecular evolutionary dynamics of *Fagopyrum* plastomes in the Polygonaceae.

Gene loss or insertion are common evolutionary phenomena and may cause variations in the plastome size [37, 38]. Moreover, the loss or insertion of the gene also influences the plastome size [41]. The *Fagopyrum* has a smaller plastome size (158,768–159,985 bp) in comparison with most of the other genera (158,981–170,232 bp) in Polygonaceae. In the plastomes of Polygonaceae, the IR region was found more conservative than the SC region, especially LSC, as most of the plastome size variations were in the LSC (Fig. 3a, Table S4). Most of the species (including *Fagopyrum* species) in Polygonaceae don’t have gene insertion. The gene insertion occurred only in a plastome of few species, i.e., *Afrobrunnichia erecta*, and *Fallopia sachalinensis* (Fig. 4, Fig S3). The increased plastome sequence length caused by gene insertion could not explain the difference of plastome sequence length between *Fagopyrum* with most of the species (excluding *Fagopyrum* species) in Polygonaceae (Fig. 4, Table S4). Hence, the smaller plastome size of *Fagopyrum* may have been caused by other factors.

In general, the short insertion/deletions (indels) lead to length variation in the plastome sequence. Although these variations occur in both the gene region and the intergenic region, they were found more in the intergenic region than gene region [42]. In the plastomes of Polygonaceae, the number of genes, as well as the length of gene regions, are mostly similar (Table 2, Fig. 3b). The length variation in the plastome sequences was evident in intergenic regions (Fig. 3b), as well as and the trend of length variation was very similar to the LSC as well as the complete plastome sequence (Fig. 3a). The results of sequence comparison among *Fagopyrum* plastomes showed a more frequent appearance of indels in the intergenic regions than the gene regions (Fig. 5). In Polygonaceae, the plastome intergenic region length was calculated for species. The intergenic region of *Fagopyrum* (44,678–45,598 bp) was found shorter than most of the other genera without IR expansion (45,174–49,747 bp). Taking the maximum difference value as an example, the longest intergenic region difference (5069 bp) between *Fagopyrum* species (*Fagopyrum leptopodum*) and other genera species (*Atraphaxis irtyschensis*) accounted for most of the maximum plastome size difference (5424 bp) between them (Table S4). These results suggest that most of the plastome size variation (5069 bp vs. 5424 bp) is caused by indels in intergenic regions when the number of genes is roughly the same.

According to the previous reports, the plastomes of Polygonaceae have a typical quadripartite structure (*Rumex* [43], (*Calligonum* [44], (*Rheum* [45]). In this study, a comparison of plastomes between *Fagopyrum* and other genera of Polygonaceae revealed the existence of similar structure (Fig. 4). Logacheva et al. [46] reported that *F. esculentum* differed from most other flowering plants at the junctions of the SSC/IRa boundary. Although, the *ycf1* gene straddled the SSC/IRa boundary in most flowering plants, the *rps15* gene straddled in the plastome of *F. esculentum* in a similar region. In this study, this boundary variation not only in *F. esculentum* but also in the plastome of *F. esculentum* subsp. *ancestrale*, *F. dibotry*3, and *F. luojishanense* (Fig. 4 type II) and occurs due to the different location of the initiation codon of the *rps15* gene from other plastomes of *Fagopyrum*. In most *Fagopyrum* plastomes, the *rps15* gene has an initiation codon in SSC, while in its variation, it was found in IRa. It could be deemed that those species which have type II boundary have evolved more than other species in *Fagopyrum*. This phenomenon may be caused by the fact that sequences adjacent to the IR/SC boundaries are more susceptible to mutations [47].

The evolutionary patterns and species relationships can be considered by the nested distribution of structural variations [39, 48]. The type III and IV IR/SC boundaries occurred in *Afrobrunnichia erecta* and *Fallopia sachalinensis,* respectively as well as their variations were caused by expansion of IRa. The IRa/LSC boundary of these two species includes an extra sequence in addition to homologous sequences of plastomes in Polygonaceae. The extra sequence in *Afrobrunnichia erecta* plastome including *rps19*, *rpl22*, *rps3*, *rpl16*, *rpl14* and their intergenic regions. The extra sequence in *Afrobrunnichia erecta* plastome including *rps19* gene (Fig. 4). The phylogenetic tree revealed the distally branching clades to be less conserved than relative basally branching clades [48]. So, it can be inferred that the IR expansion of *Afrobrunnichia erecta* and *Fallopia sachalinensis* plastome is caused by the retention of common ancestral characteristics, and gene loss may have occurred in most species (including *Fagopyrum* species) of Polygonaceae. However, the inference is based on the comparison of a single plastome of *Afrobrunnichia* and *Fallopia* with other Polygonaceae plastomes. Therefore, future research with more samples is needed to determine whether the gene insertion of the IR region is common in *Afrobrunnichia* and *Fallopia*.

Moreover, detecting the highly informative and variable genome regions can be important for diagnostic genetic marker development and DNA barcoding [27]. In *Fagopyrum,* plastome regions *rbcL*, *accD*, *trnK (UUU)*, *trnC (GCA)*-*rpoB*, *matK*, *trnH-psbA*, *trnL*, *psbE-psbL,* and *ndhA* intron have been widely used to construct the phylogeny at the species level, which is often supplemented with nrDNA ITS and/or nuclear gene sequences (*FLO/LFY*, *AG*) [12, 15–17, 19, 49, 50]. In general, there is a greater variation in intergenic regions than in gene regions, as has also been observed in this study (Table S6). Although to increase the sequence variation and obtain considerable support, some intergenic regions have also been used for phylogenetic analysis. The rapid rate of evolution of intergenic regions may lead to topology confusion. This study demonstrates that phylogenetic analysis based on gene regions concatenation is more comparable to the complete plastome than intergenic regions concatenation (Fig. S5). It shows that the Pi of the gene regions is more appropriate for species identification than intergenic regions. Therefore, to reconstruct the phylogenetic tree of *Fagopyrum*, the combination of the top eight gene regions (*rps15*, *trnK*, *trnL*, *matK*, *ndhA*, *clpP*, *rpoC2*, *rpl16*) were considered as an economical and accurate candidate marker. To explore the species relationship of *Fagopyrum,* in addition to DNA loci and their combinations, repeats or SNPs were also be used as molecular markers [51, 52]. The number of different types of repeat sequences in *Fagopyrum* plastomes differs in different groups (Fig. 6), as well as identity of plastomes also differed significantly between intra-groups and inter-groups (Fig. 7). These results suggest that sequence diversity also reveals either distant or close relationships between species. However, unfortunately, these differences were not found significant between subgroups. Thus, based on the mutation hotspot and the complete plastome sequence, phylogenetic analysis is still the best way to determine the relationship between *Fagopyrum* species.

### Phylogenomic inferences and species relationships

A well-supported phylogenomic framework was reconstructed based on 12 newly sampled plastomes included in the present study as well as eight plastomes reported in previous studies [35, 36, 53, 54]. The monophyly and group division based on DNA loci was also found consistent with previous studies in *Fagopyrum* [12, 15–17, 25, 55, 56]. The urophyllum group consisted the three subgroups, including *F. leptopodum-F. statice* subgroup, *F. capillatum*-*F. gracilipes* subgroup, and *F. callianthum-F. pleioramosum* as reported in the previous studies. However, the relationship among these subclades has been inconsistent in different studies, and new species were rarely included. The current study resolves the uncertain systematic position of these subgroups. The subclade I (*F. leptopodum*, *F. leptopodum* var. *grossii*, and *F. statice*) was found closely related to the subclade II (F. *gracilipes*, *F. gracilipes* var. *odontopterum*, and *F. luojishanense*) followed by the non-monophyletic species *F. urophyllum* (*F. urophyllum* (liana), *F.* sp, and *F. urophyllum*) and subclade IV (*F. caudatum* and *F. qiangcai*) (Fig. 8).

The well-supported phylogenomic framework improves our understanding of species relationships within group of *Fagopyrum*. Additionally, this study also provides insights into the new species described in recent years. In subclade I, *F. statice* sister to *F. leptopodum* and *F. leptopodum* var. *grossii.* Species with smaller achenes are a character of subclade I. The subclade II includes a recently described species, i.e., *F. luojishanense* [23], which was morphologically most similar to *F. gracilipes.* However, due to the lack of plastome data on members of the urophyllum group, the systematic position of *F. luojishanense* remains unclear [36]. Our phylogenetic tree indicated that *F. luojishanense* belonged to the urophyllum group and was closely related to *F. gracilipes* (Fig. 8). The subclade III includes woody shrub *F. urophyllum*, scandent shrub *F. urophyllum* (liana) and *F.* sp (intermediate form between liana and woody), respectively. Species *F. urophyllum* was previously reported as non-monophyletic based on nuclear and chloroplast DNA sequences [49], in consistent with our study (Fig. 8). *F. urophyllum* is generally woody shrubs, but we detected a liana scandent shrub population in the wild, that is *F.* sp. Based on the non-monophyletic topology and wild population with intermediate form between liana and woody found in *F. urophyllum*, we infer that incomplete lineage sorting and/or hybridization occurred in this species.

The subclade IV included *F. caudatum*, as well as the newly described *F. qiangcai*, which has been considered closely related to *F. esculentum* based on morphology [22] or to *F. leptopodum* based on molecular data [12]. In this study, *F. qiangcai* was taken as a sample from a population with leafy bases, leaves with bright red veins, and white punctate adaxial leaf surfaces from Lixian, Sichuan Province (Fig. 1g). The morphological characteristics of samples were also similar to the *F. callianthum,* which was reported by Ohsako and Ohnishi [19]. Tang et al. [3] considered *F. callianthum* as a synonym of *F. qiangcai*. Another sample *F. caudatum* was considered has two other synonyms, *F. pleioramosum* and *F. wenchuanense* [3]. Based on both plastome and nuclear DNA loci, *F. pleioramosum* showed more closeness to *F. qiangcai* (*F. callianthum*) [15, 16, 18–20, 49]. On the other hand, *F. wenchuanense* were found closely related to *F. gracilipes,* based on the morphology [22] and molecular data [12], respectively. The previous phylogenetic analyses were unable to resolve the relationship between *F. caudatum* (*F. pleioramosum/F. wenchuanense*) and other species based on a small number of DNA loci. Combining the results of previous studies and phylogenetic analysis in this study, we determined that the close relationship between *F. caudatum* (*F. pleioramosum/F. wenchuanense*) and *F. qiangcai* (*F. callianthum*) and confirmed the systematic position of this subclade at the base of urophyllum group (Fig. 8).

The plastome phylogenomic provided strong support for relationships between subclades in the cymosum group. The reconstructed subclade V (*F. megaspartanium*, *F. cymosum*, *F. dibotrys*1, *F. dibotrys*2, and *F. dibotrys*3) was found more closely related to subclade VI (*F. tataricum* and *F. tataricum* (cultivar Miqiao) than subclade VII (*F. esculentum* and *F. esculentum* subsp. *ancestrale*) (Fig. 8), like findings of previous studies [4, 15–17]. However, the taxonomic status of *F. megaspartanium* and *F. cymosum* is still a controversial issue. In addition, *F. megaspartanium* was treated as a variety or a closely related species of *F. cymosum* [17, 57]*.* According to Ohsako and Li [4], in a phylogenetic analysis based on *matK* sequences, *F. cymosum* appeared to be a complex species with two branches. The report of Chen [58] suggests that *F. megaspartanium* might be an ancestor of the cultivated buckwheat species viz., *F. esculentum,* and *F. tataricum*. Furthermore, it was suggested that the *F. esculentum* subsp. *ancestrale* might be a hybrid species between *F. cymosum* and *F. esculentum* [59], and there is a complex evolutionary history among the *F. cymosum* species. In this study, the phylogenetic analysis based on plastome sequences strongly supported the two branches of the complex species *F. cymosum,* i.e., one branch including our samples *F. cymosum*, *F. dibotrys*1, and *F. megaspartanium*, while the other comprised of the samples *F. dibotrys*2 and *F. dibotrys*3 (Fig. 8). Therefore, we agree that *F. cymosum* species complex have with two branches. And we speculate that *F. megaspartanium* and *F. cymosum* belong to the same branch, and *F. megaspartanium* is the representative of this branch based on the phylogenomic tree of this study. Furthermore, to clarify the other branches of the *F. cymosum* species complex, further sampling of the subclades V is needed in future studies.

The phylogenetic framework based on plastomes not only elucidated the taxonomic relationships but also enhanced our understanding of morphological characteristics of *Fagopyrum*. Achene size is considered one of the key taxonomic characteristics of *Fagopyrum* [2]. In this study, the achene size show difference between the urophyllum group and cymosum group in the phylogenetic tree. Most of the achenes of species in the urophyllum group are less than 5 mm and gradually become smaller from the basal branch (*F. caudatum-F. qiangcai* subclade) to the distal branch (*F. leptopodum-F. statice* subclade). On the contrary, in the cymosum group, the achenes of species are mostly greater than 5 mm and gradually increase from the basal branch (*F. esculentum* subclade) to the distal branch (*F. megaspartanium-F. cymosum* subclade) (Fig. 8). The results suggest that these two groups are likely to have a common ancestor with an achene size of around 5 mm and evolved in different directions, resulting in the present achene size differences. Thus, based on phylogenetic trees, it can be inferred that the evolution of morphological characters facilitates classification and species identification.

## Conclusions

This study provides a detailed comparison of plastome characteristics in *Fagopyrum* and a phylogenomic framework of *Fagopyrum* with strongly support. The plastome size difference, which is mainly due to deletions of intergenic regions, shows that the *Fagopyrum* plastome has been conserved within genera and remains specific between genera. The IR/SC boundary variations, which mainly occur due to gene loss, revealed the evolutionary dynamics of *Fagopyrum* in Polygonaceae. In *Fagopyrum,* the plastome sequence diversification demonstrates its power in resolving evolutionary relationships. It is possible to reconstruct phylogenetic relationships with high support even from several gene sequences. Moreover, the number of repeats in plastomes and relatively low sequence similarity between groups of *Fagopyrum* allows the development of molecular markers for species identification based on SNPs and repeats. As demonstrated in this study, plastome sequences can reveal species relationships and evolutionary changes in taxa. In addition to appending new genomic resources, these findings will be useful for future studies of the evolution and phylogeny of *Fagopyrum*.

## Methods

### Taxon sampling and DNA sequencing

A total of 12 wild individuals representing the eight species and two varieties were collected and sequenced (Fig. 1, Table S1). The formal identification of plants was performed according to the Flora of China [1]. The names of the species, locations of the specimen collections, voucher numbers, and GenBank accession numbers for all samples used in this study are listed in Table S1. All the samples were identified by An-hu Wang (Panxi Crops Research and Utilization Key Laboratory of Sichuan Province, Xichang University) and Qing-fu Chen (Research Center of Buckwheat Industry Technology, Guizhou Normal University) based on the morphological characters and the species were preserved in the herbarium of Panxi Crops Research and Utilization Key Laboratory of Sichuan Province. The fresh leaf samples were collected and dried using silica gel. To extract the total DNA from collected samples, a modified Cetyltrimethylammonium Bromide (CTAB) method was used [60]. The sequencing was performed on the Illumina NovaSeq 6000 platform at TSINGKE Biological Technology Co., Ltd. (Beijing, China) using the paired-end 150 bp reads with an average insert size of 300–400 bp. Additionally, 57 plastomes of Polygonaceae (including eight plastomes of *Fagopyrum*) and two plastomes of Plumbaginaceae were downloaded for the phylogenetic analysis and sequence comparison. The GenBank accession numbers for taxa sampled in this study are listed in Table S2.

### Plastome assembly and annotation

The quality check of raw reads was performed using FastQC v0.11.9. The plastomes were de novo assembled using GetOrganelle v1.6.2 [61] considering the plastomes of *F. dibotrys* (KY275181) and *F. luojishanense* (KY275182) as references. To verify the sequencing depth and overlapping contigs, the cleaned reads were mapped to the reference plastomes using Geneious R11.0.5 [62] (Table 1). The Plastid Genome Annotator (PGA) was used to annotate the plastomes [63], followed by the validation of annotation using GeSeq [64] (Table 2). A circular map of the plastomes was generated employing the Organellar Genome DRAW (OGDRAW) [65] (Fig. 2).

### Plastome comparative analysis

The guanine-cytosine content (GC content) of each accession was determined in Geneious. The sequence divergence among 12 plastomes was visualized using the mVISTA program [66], considering *F. gracilipes* as a reference (Fig. S1). Since the large single-copy (LSC) and small single-copy (SSC) regions were flanked by the two inverted repeat (IR) regions, the junctions between these regions were found by calculating the IR region length using REPuter [67] (Fig. S2, S3). *Fagopyrum* plastomes were characterized using the Multiple Alignment using Fast Fourier Transform (MAFFT) algorithm [68], and the checked sequence similarity/identity was assessed in Geneious (Fig. 3, 8, Table S4). The sequence variation of *Fagopyrum* plastomes was mapped by dividing the gene sequences and the intergenic sequences without IRa regions into overlapping and non-overlapping matrices of 500 and 250 bp, respectively. To calculate the phylogenetic informativeness (Pi), insertions/deletions (indels), and single nucleotide polymorphisms (SNPs), DnaSP was employed [69] (Fig. 5).

The protein-coding genes were used to evaluate the evolutionary rate of the different genes within *Fagopyrum*. We calculated the rate of non-synonymous substitution (Ka), synonymous substitutions (Ks) and their ratio (Ka/Ks). *F. urophyllum*, a sample that is basal to genus *Fagopyrum*, was used as a reference and protein-coding genes of all the species were aligned with *F. urophyllum* by Muscle pairwise alignment in Geneious and analysed in DnaSP for Ka and Ks without stop codon (Fig. S4, Table S5).

### Repeats analysis

Using the online program REPuter [66], SDRs were identified, including four types, i.e., forward repeats, reverse repeats, complement repeats, and palindromic repeats (Fig. 6a, b). The parameters were set as (1) Hamming distance to 3; (2) 90% or greater sequence identity; and (3) minimal repeat size of 30 bp. SSRs were also identified via Perl script MISA [70], including mono, di, tri, tetra, penta, and hexanucleotides. The minimum numbers of the SSRs were set to 10, 5, 4, 3, 3, and 3 for mono, di, tri, tetra, penta, and hexanucleotides, respectively (Fig. 6c). An online program, Tandem Repeats Finder [71], was employed to find the tandem repeats, where the similarity percentage of two repeat copies was at least 90% as well as the minimal repeat size was 10 bp (Fig. 6d).

### Phylogenetic analysis and hotspots identification

The complete plastome sequences were used for phylogenetic studies of Polygonaceae and *Fagopyrum*. To investigate the systematic position of *Fagopyrum* in the family Polygonaceae, 69 plastome data sets encompassing a wide phylogenetic diversity in Polygonaceae were included in analyses. *Limonium sinense* and *Plumbago auriculata* from the family Plumbaginaceae were used to root the phylogenetic tree (Fig. 4). To investigate relationships among the species within the genus *Fagopyrum*, the phylogenetic analyses were performed using 20 plastomes. *Afrobrunnichia erecta* and *Persicaria chinensis* from the family Polygonaceae were used for rooting the phylogenetic tree (Fig. 8). Phylogenetic inference of Polygonaceae was conducted using the maximum likelihood (ML) method. Moreover, phylogenetic inference of *Fagopyrum* was conducted using three approaches, including ML, maximum parsimony (MP), and Bayesian inference (BI).

The ML analysis was performed using RAxMLHPC2 v8.0 [72] with the GTR + GAMMA nucleotide substitution model on the CyberInfrastructure for Phylogenetic Research (CIPRES) Science Gateway v3.3 [73]. The analysis of 1,000 rapid bootstrap replicates (-x) was followed by a search for the best-scoring ML tree in one single program run (-f a). The MP analysis was carried out in PAUP* v4.0 b10 [74] with equally weighted and unordered characters. Searches were performed on 100 replicates of random taxon addition using tree-bisection-reconnection (TBR) branch swapping with the MulTrees option. Bootstrap analyses, including parsimony bootstrap percentages (PBP), and 1,000 pseudoreplicates, were carried out with the same parameters to examine the relative level of clade support. BI analyses were performed using MrBayes v3.2 [75]. Tree searches with a randomly chosen starting tree were run for each dataset consisting of one million generations, with sampling every 100 generations. An initial 25% of sampled trees were discarded. The posterior probability of values was calculated from the remaining trees. Stationarity was reached when the average standard deviation of split frequencies was below 0.01.

To explore the informative regions of the plastome, six datasets consisting of the following sequences were included in phylogenetic analyses using three different methods, namely ML, MP, and BI: (a) all gene regions; (b) all intergenic regions; (c) the top 20 hotspots of gene regions; (d) the top 20 hotspots of intergenic regions; (e) the top eight hotspots of gene regions; and (f) the top eight hotspots of intergenic regions (Fig. S5, Table S6).

## Supplementary Information

**Additional file 1: Figure S1.** Visualized alignment of the *Fagopyrum* plastomes. The mVISTA-based identity plots show the sequence identity among the plastomes; sawtooth indicates the sequence difference. *F*. *gracilipes* was used as a reference. Coding and noncoding-regions are colored blue and red, respectively. **Figure S2.** Comparative analysis of junction sites in *Fagopyrum*. **Figure S3.** Comparative analysis of junction sites in nine Polygonaceae genera. **Figure S4.** The rate of synonymous (Ks) and non-synonymous (Ka) substitutions protein-coding genes of the *Fagopyrum* plastomes. **a** The Ka/ Ks of urophyllum group species plastome genes. **b** The Ka/ Ks of cymosum group species plastome genes. **Figure S5.** Phylogenetic relationships of *Fagopyrum* based on plastome gene regions and intergenic regions. 20 gene regions and 20 intergenic regions used in the phylogenetic tree mentioned in Table S5. Values on the left are posterior probabilities (PP), and middle and right are the bootstrap support (BS) percentages from the maximum likelihood analyses and maximum parsimony; * indicates PP=1 and BS=100%; - indicates BS < 50%. The colored branches in the phylogenetic tree indicate differences from the tree based on complete plastome sequences. **Table S1**. Voucher information for *Fagopyrum* specimens in this study. **Table S2.** Plastome sequences downloaded from GenBank. **Table S3.** Plastome gene content and functional classification in *Fagopyrum*. **Table S4.** Plastomes characteristics of Polygonaceae species. **Table S5.** The rate of synonymous (Ks) and non-synonymous (Ka) substitutions protein-coding genes of the *Fagopyrum* plastomes. **Table S6.** Hotspots (gene/intergenic regions) among *Fagopyrum *plastomes.

**Additional file 2. **Plastome DNA Sequences of Fagopyrum.

## Data Availability

All sequences used in this study are available from the National Center for Biotechnology Information (NCBI) (see Additional file 1: Table S1) and from Supplementary Information (Additional file 2: Plastome DNA sequences of *Fagopyrum*). The GenBank accession numbers are MZ491847 and MZ702791–MZ702801. All sequences will be made public after this manuscript is published or after July 2, 2023.
