# Supplementary material for: Plastome comparison and phylogenomics of Fagopyrum (Polygonaceae): insights into sequence differences between Fagopyrum and its related taxa
Source: BMC Plant Biol. 2022 Jul 14;22:339. doi: 10.1186/s12870-022-03715-5 (PMC9281083; doi:10.1186/s12870-022-03715-5)
Supplement: Supplementary file 1 — Additional file 1: Figure S1. Visualized alignment of the Fagopyrum plastomes. The mVISTA-based identity plots show the sequence identity among the plastomes; sawtooth indicates the sequence difference. F. gracilipes was used as a reference. Coding and noncoding-regions are colored blue and red, respectively. Figure S2. Comparative analysis of junction sites in Fagopyrum. Figure S3. Comparative analysis of junction sites in nine Polygonaceae genera. Figure S4. The rate of synonymous (Ks) and non-synonymous (Ka) substitutions protein-coding genes of the Fagopyrum plastomes. a The Ka/ Ks of urophyllum group species plastome genes. b The Ka/ Ks of cymosum group species plastome genes. Figure S5. Phylogenetic relationships of Fagopyrum based on plastome gene regions and intergenic regions. 20 gene regions and 20 intergenic regions used in the phylogenetic tree mentioned in Table S5. Values on the left are posterior probabilities (PP), and middle and right are the bootstrap support (BS) percentages from the maximum likelihood analyses and maximum parsimony; * indicates PP=1 and BS=100%; - indicates BS < 50%. The colored branches in the phylogenetic tree indicate differences from the tree based on complete plastome sequences. Table S1. Voucher information for Fagopyrum specimens in this study. Table S2. Plastome sequences downloaded from GenBank. Table S3. Plastome gene content and functional classification in Fagopyrum. Table S4. Plastomes characteristics of Polygonaceae species. Table S5. The rate of synonymous (Ks) and non-synonymous (Ka) substitutions protein-coding genes of the Fagopyrum plastomes. Table S6. Hotspots (gene/intergenic regions) among Fagopyrum plastomes. [file 12870_2022_3715_MOESM1_ESM.docx]

**Supplementary materials**

**Figure S1 Visualized alignment of the *Fagopyrum*** **plastomes.** The mVISTA-based identity plots show the sequence identity among the plastomes; sawtooth indicates the sequence difference. *F. gracilipes* was used as a reference. Coding and noncoding-regions are colored blue and red, respectively.

**Figure S2 Comparative analysis of junction sites in** ***Fagopyrum*.**

**Figure S3 Comparative analysis of junction sites in** **nine Polygonaceae genera.**

**Figure S4 The rate of synonymous (Ks) and non-synonymous (Ka) substitutions protein-coding genes of the *Fagopyrum* plastomes. a** The Ka/ Ks of urophyllum group species plastome genes. **b** The Ka/ Ks of cymosum group species plastome genes.

**Figure S5 Phylogenetic relationships of *Fagopyrum* based on plastome gene regions and intergenic regions.** 20 gene regions and 20 intergenic regions used in the phylogenetic tree mentioned in **Table S5**. Values on the left are posterior probabilities (PP), and middle and right are the bootstrap support (BS) percentages from the maximum likelihood analyses and maximum parsimony; * indicates PP=1 and BS=100%; - indicates BS < 50%. The colored branches in the phylogenetic tree indicate differences from the tree based on complete plastome sequences.

**Table S1.** Voucher information for *Fagopyrum* specimens in this study.

**Table S2.** Plastome sequences downloaded from GenBank.

**Table S3.** Plastome gene content and functional classification in *Fagopyrum.*

**Table S4.** Plastomes characteristics of Polygonaceae species.

**Table S5.** The rate of synonymous (Ks) and non-synonymous (Ka) substitutions protein-coding genes of the *Fagopyrum* plastomes.

Table S6 Hotspots (gene/intergenic regions) among *Fagopyrum* plastomes.


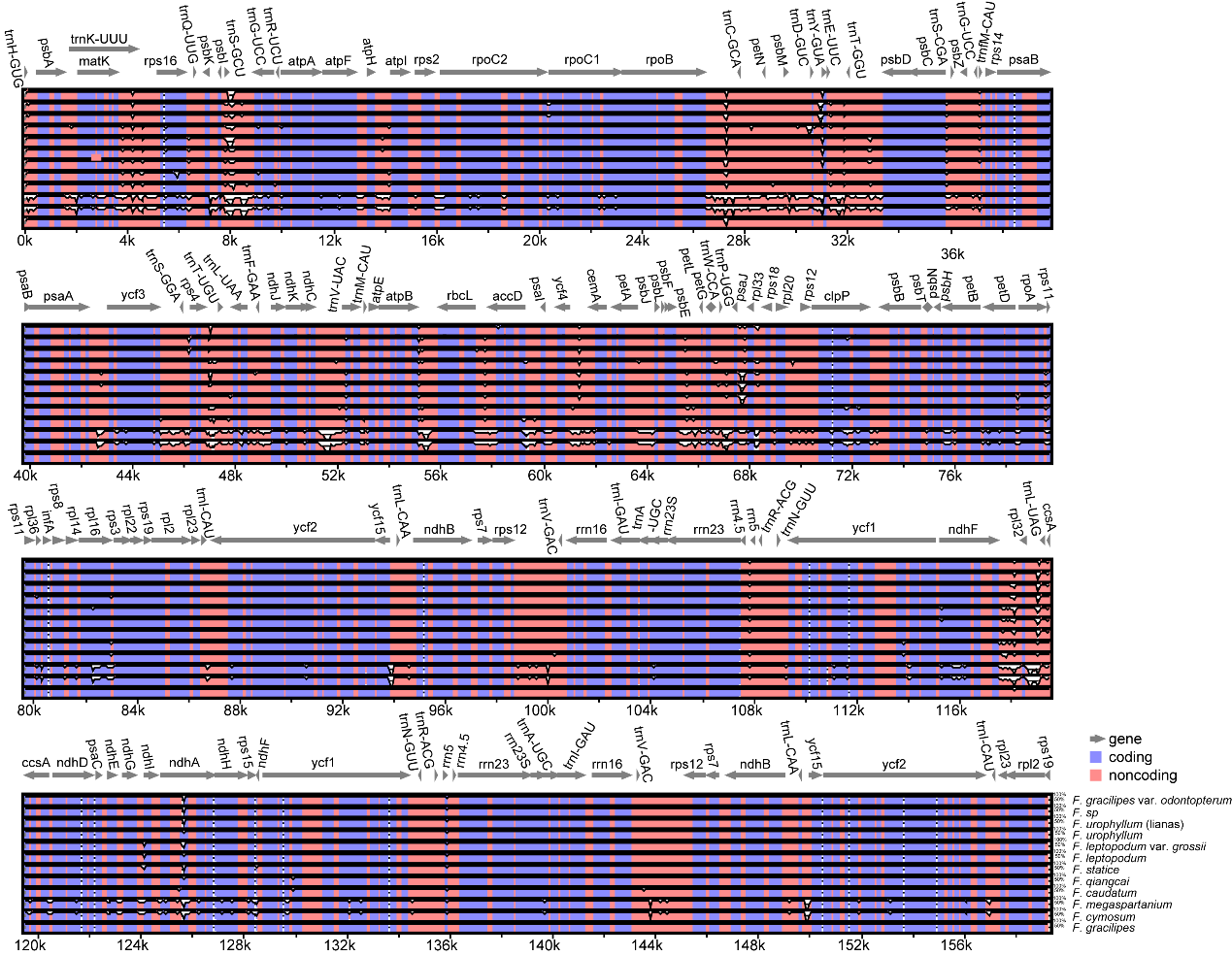


**Figure S1**


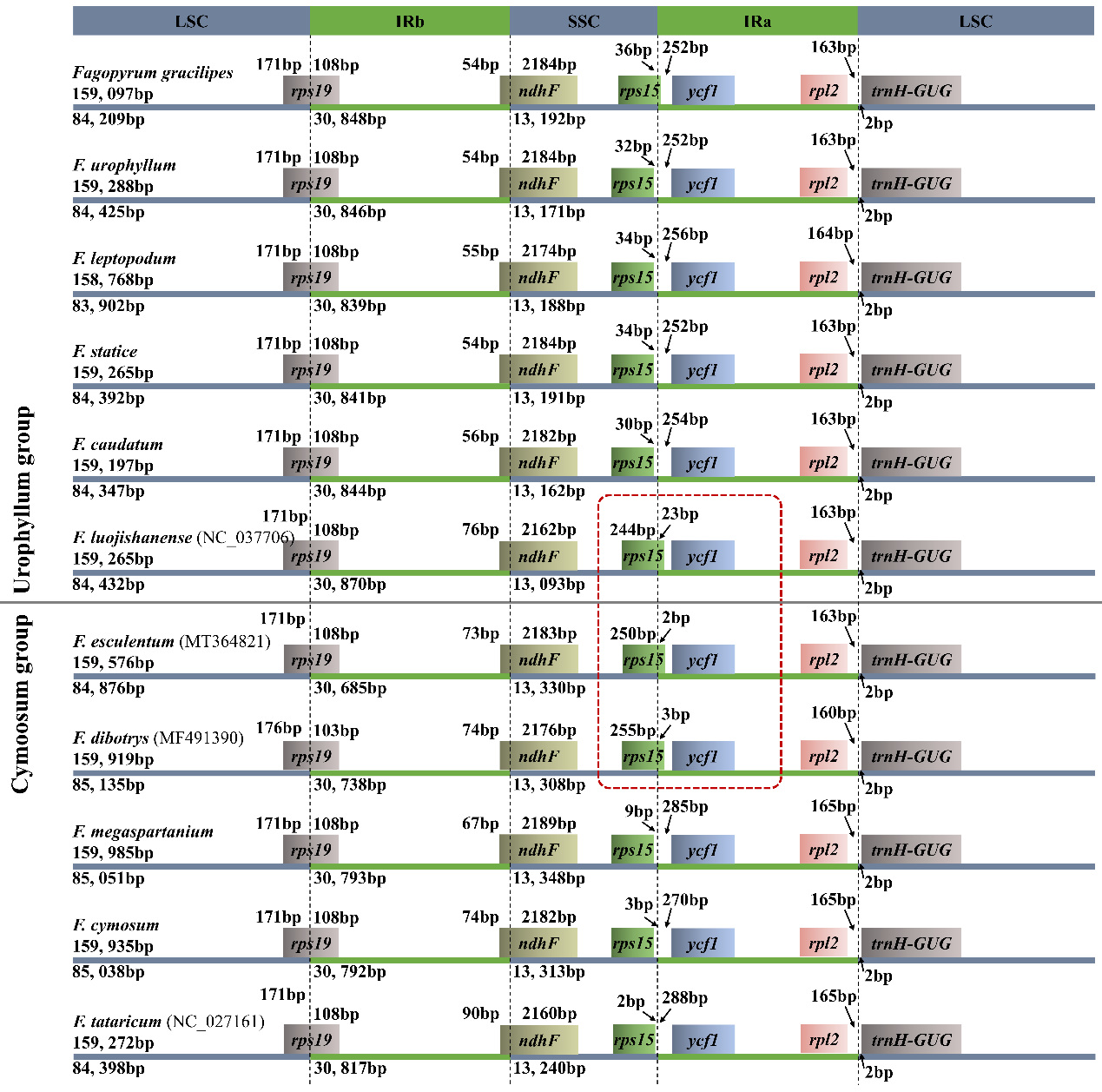


**Figure S2**


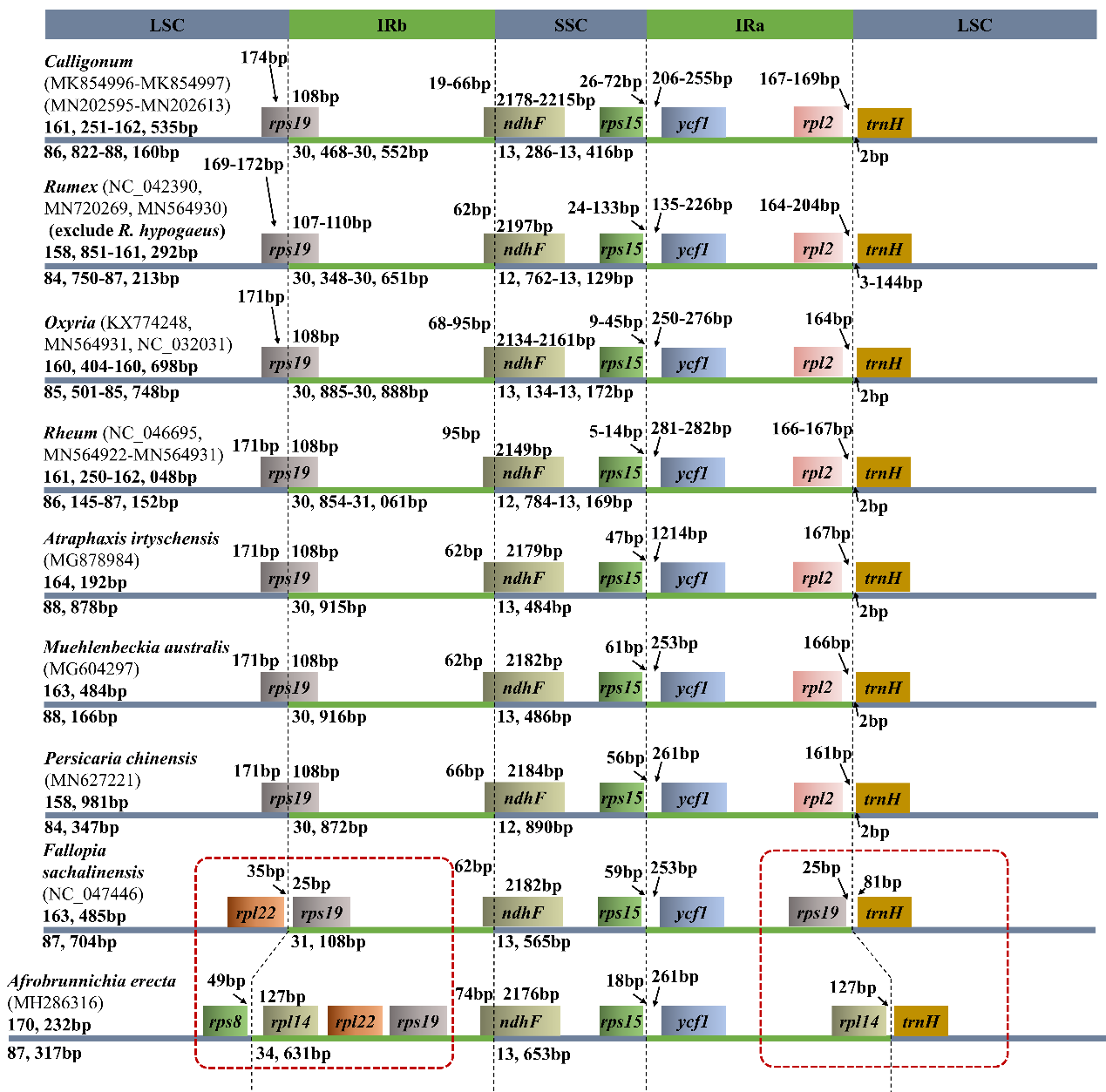


**Figure S3**


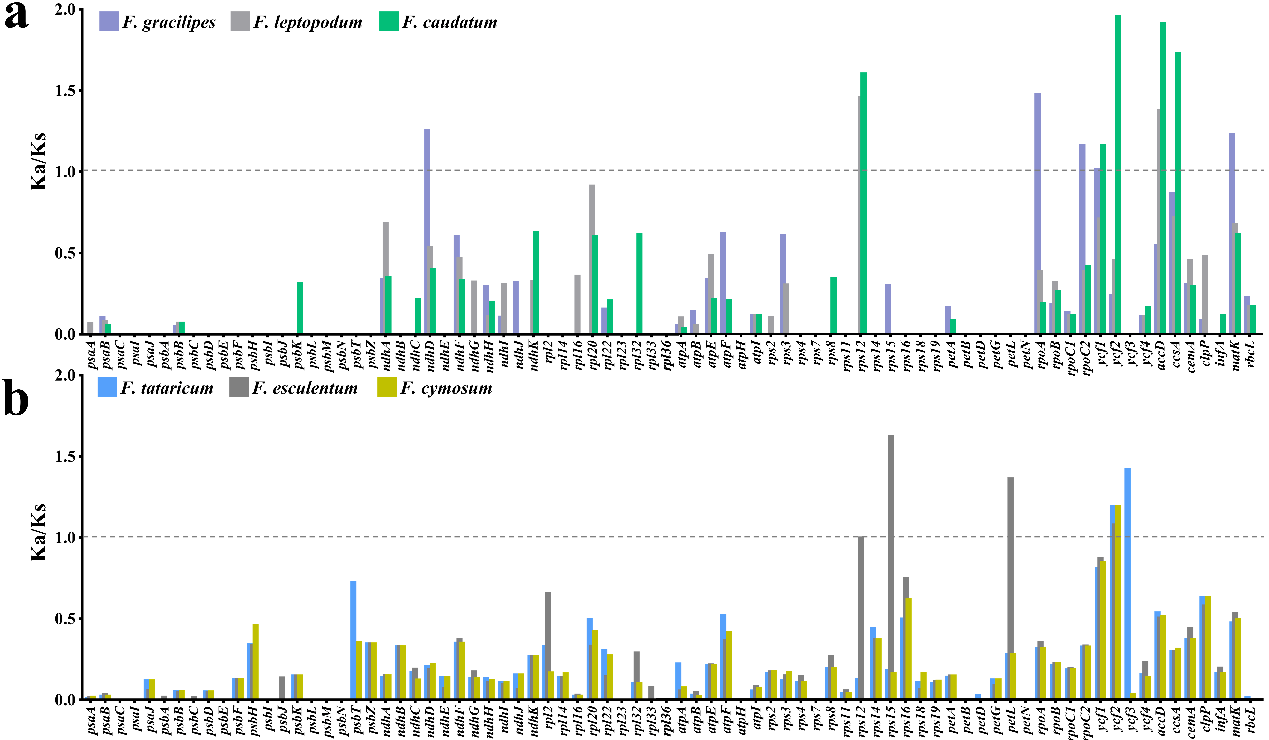


**Figure S4**


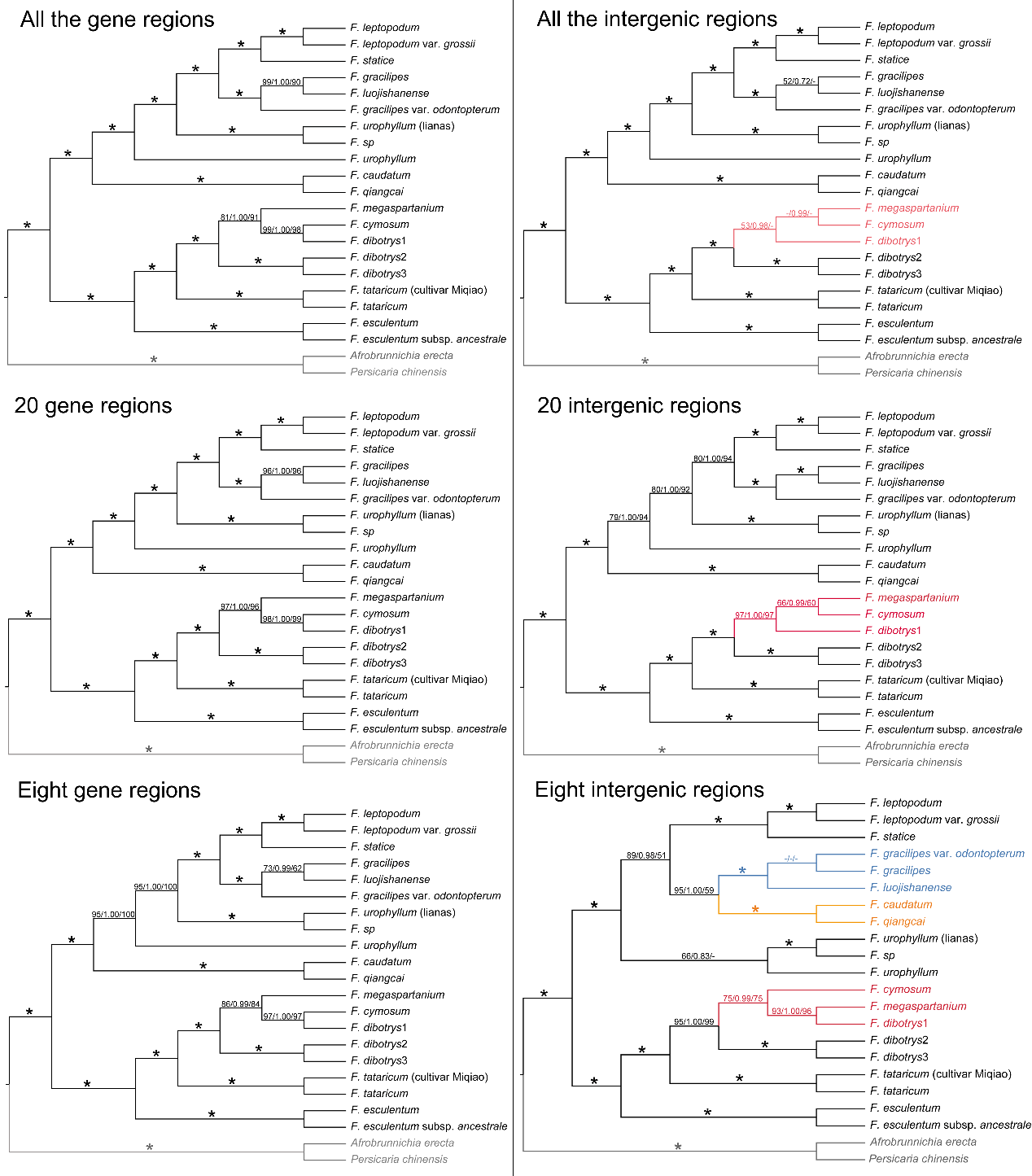


**Figure S5**

**Table S1** Voucher information for *Fagopyrum* specimens in this study.

| **Taxon** | **Species** | **NO.** | **Location** | **Latitude** | **Longitude** | **date/Voucher** | **Accession** |
| --- | --- | --- | --- | --- | --- | --- | --- |
| cymosum group | *F. cymosum* | 818 | Yanyuan, Sichuan | 27°20′17.2″ | 101°11′46.4″ | 2019-10-30 | MZ702796 |
|  | *F. megaspartanium* | 815 | Puge, Sichuan | 27°35′37.8″ | 102°26′23.3″ | 2019-10-29 | MZ702795 |
| urophyllum group | *F. caudatum* | 837 | Wenchuan, Sichuan | 31°33′42.6″ | 103°25′8.1″ | 2020-10-13 | MZ702799 |
|  | *F. gracilipes* var. *odontopterum* | 801 | Leibo, Sichuan | 28°17′10.4″ | 103°36′16.5″ | 2019-10-26-3 | MZ491847 |
|  | *F. gracilipes* | 802 | Leibo, Sichuan | 28°17′10.4″ | 103°36′16.5″ | 2019-10-26-4 | MZ702791 |
|  | *F. leptopodum* | 822 | Muli, Sichuan | 27°46′59.5″ | 101°13′2.7″ | 2019-10-31-2 | MZ702797 |
|  | *F. leptopodum var. grossii* | 821 | Muli, Sichuan | 27°46′59.5″ | 101°13′2.7″ | 2019-10-31-1 | MZ702798 |
|  | *F. qiangcai* | 837 | Lixian, Sichuan | 31°29′28.9″ | 103°12′36.6″ | 2020-10-14 | MZ702800 |
|  | *F. sp* | 804 | Leibo, Sichuan | 28°18′23.9″ | 103°38′1.1″ | 2019-10-26-1 | MZ702793 |
|  | *F. statice* | 867 | Shilin, Yunnan | 24°37′43.4″ | 103°34′14.2″ | 2020-11-10 | MZ702801 |
|  | *F. urophyllum* (lianas) | 813 | Puge, Sichuan | 27°32′15.6″ | 102°37′12.1″ | 2019-10-28 | MZ702794 |
|  | *F. urophyllum* | 803 | Leibo, Sichuan | 28°17′55.1″ | 103°36′33.5″ | 2019-10-26-2 | MZ702792 |

**Table S2** Plastome sequences downloaded from Genebank.

| **Species** | **In/Out group** | | **Family** | **Genus** | **GenBank Accession** | **References** |
| --- | --- | --- | --- | --- | --- | --- |
| *Calligonum arborescens* | Ingroup | | Polygonaceae | *Calligonum* | NC_049140/MN202599 | [1] |
| *Calligonum caput-medusae* | Ingroup | | Polygonaceae | *Calligonum* | NC_049141/MN202600 | [1] |
| *Calligonum caput-medusae2* | Ingroup | | Polygonaceae | *Calligonum* | MN202601 | [1] |
| *Calligonum junceum1* | Ingroup | | Polygonaceae | *Calligonum* | NC_049147/MN202609 | [1] |
| *Calligonum junceum4* | Ingroup | | Polygonaceae | *Calligonum* | MK854996 | [1] |
| *Calligonum junceum3* | Ingroup | | Polygonaceae | *Calligonum* | MN202610 | [1] |
| *Calligonum aphyllum2* | Ingroup | | Polygonaceae | *Calligonum* | MN202596 | [1] |
| *Calligonum aphyllum1* | Ingroup | | Polygonaceae | *Calligonum* | NC_049137/MN202595 | [1] |
| *Calligonum densum* | Ingroup | | Polygonaceae | *Calligonum* | NC_049144/MN202604 | [1] |
| *Calligonum cordatum* | Ingroup | | Polygonaceae | *Calligonum* | NC_049143/MN202603 | [1] |
| *Calligonum juochiangense* | Ingroup | | Polygonaceae | *Calligonum* | NC_049138/MN202597 | [1] |
| *Calligonum korlaense1* | Ingroup | | Polygonaceae | *Calligonum* | NC_049149/MN202612 | [1] |
| *Calligonum korlaense2* | Ingroup | | Polygonaceae | *Calligonum* | MN202613 | [1] |
| *Calligonum klementzii* | Ingroup | | Polygonaceae | *Calligonum* | NC_049148/MN202611 | [1] |
| *Calligonum colubrinum 2* | Ingroup | | Polygonaceae | *Calligonum* | NC_049142/MN202602 | [1] |
| *Calligonum gobicum* | Ingroup | | Polygonaceae | *Calligonum* | NC_049139/MN202598 | [1] |
| *Calligonum ebinuricum3* | Ingroup | | Polygonaceae | *Calligonum* | MN202606 | [1] |
| *Calligonum ebinuricum2* | Ingroup | | Polygonaceae | *Calligonum* | MN202607 | [1] |
| *Calligonum ebinuricum4* | Ingroup | | Polygonaceae | *Calligonum* | MN202605 | [1] |
| *Calligonum ebinuricum1* | Ingroup | | Polygonaceae | *Calligonum* | NC_049145 | [1] |
| *Calligonum jeminaicum* | Ingroup | | Polygonaceae | *Calligonum* | NC_049146/MN202608 | [1] |
| *Calligonum junceum2* | Ingroup | | Polygonaceae | *Calligonum* | MK854997 | [1] |
| **Table S2 (continued)** |  | |  |  |  |  |
| **Species** | **In/Out group** | | **Family** | **Genus** | **GenBank Accession** | **References** |
| *Rumex wittrockii* | Ingroup | | Polygonaceae | *Rheum* | NC_035950/KY985269 | [2] |
| *Rumex crispus1* | Ingroup | | Polygonaceae | *Rheum* | MN564930 | - |
| *Rumex crispus2* | Ingroup | | Polygonaceae | *Rheum* | MN055629 | - |
| *Rumex japonicus2* | Ingroup | | Polygonaceae | *Rheum* | MK058527 | [3] |
| *Rumex japonicus1* | Ingroup | | Polygonaceae | *Rheum* | MN720269 | - |
| *Rumex acetosa* | Ingroup | | Polygonaceae | *Rheum* | NC_042390/MH359405 | [4] |
| *Rumex hypogaeus* | Ingroup | | Polygonaceae | *Rheum* | NC_050054/MT017652 | [5] |
| *Oxyria digyna* | Ingroup | | Polygonaceae | *Oxyria* | MN564931 | [2] |
| *Oxyria sinensis1* | Ingroup | | Polygonaceae | *Oxyria* | NC_032031 | - |
| *Oxyria sinensis2* | Ingroup | | Polygonaceae | *Oxyria* | KX774248 | - |
| *Rheum pumilum* | Ingroup | | Polygonaceae | *Rheum* | MN564927 | [2] |
| *Rheum przewalskyi* | Ingroup | | Polygonaceae | *Rheum* | MN564926 | [2] |
| *Rheum nobile* | Ingroup | | Polygonaceae | *Rheum* | MN880789 | [6] |
| *Rheum acuminatum1* | Ingroup | | Polygonaceae | *Rheum* | MN564922 | [2] |
| *Rheum acuminatum2* | Ingroup | | Polygonaceae | *Rheum* | MN514858 | [6] |
| *Rheum racemiferum1* | Ingroup | | Polygonaceae | *Rheum* | MN564928 | [2] |
| *Rheum tanguticum2* | Ingroup | | Polygonaceae | *Rheum* | NC_046695/MK674897 | [2] |
| *Rheum tanguticum1* | Ingroup | | Polygonaceae | *Rheum* | MN564929 | [2] |
| *Rheum officinale* | Ingroup | | Polygonaceae | *Rheum* | MN564925 | [2] |
| *Rheum palmatum* | Ingroup | | Polygonaceae | *Rheum* | NC_027728/KR816224 | [7] |
| *Rheum hotaoense* | Ingroup | | Polygonaceae | *Rheum* | MN564924 | [2] |
| *Rheum franzenbachii* | Ingroup | | Polygonaceae | *Rheum* | MN564923 | [2] |
| **Table S2 (continued)** |  | |  |  |  |  |
| **Species** | **In/Out group** | | **Family** | **Genus** | **GenBank Accession** | **References** |
| *Atraphaxis irtyschensis* | Ingroup | | Polygonaceae | *Atraphaxis* | MG878984 | - |
| *Fallopia sachalinensis* | Ingroup | | Polygonaceae | *Fallopia* | NC_047446/MK842154 | [8] |
| *Muehlenbeckia australis* | Ingroup | | Polygonaceae | *Muehlenbeckia* | MG604297 | [9] |
| *Fagopyrum luojishanense* | Ingroup | | Polygonaceae | *Fagopyrum* | NC_037706 | [10] |
| *Fagopyrum dibotrys1* | Ingroup | | Polygonaceae | *Fagopyrum* | NC_037705/KY275181 | [10] |
| *Fagopyrum dibotrys2* | Ingroup | | Polygonaceae | *Fagopyrum* | MH196562 | [11] |
| *Fagopyrum dibotrys3* | Ingroup | | Polygonaceae | *Fagopyrum* | MF491390 | - |
| *Fagopyrum tataricum* | Ingroup | | Polygonaceae | *Fagopyrum* | NC_027161/KM201427 | [12] |
| *Fagopyrum tataricum* (cultivar Miqiao) | | Ingroup | Polygonaceae | *Fagopyrum* | KX085498 | [13] |
| *Fagopyrum esculentum* subsp. *ancestrale* | | Ingroup | Polygonaceae | *Fagopyrum* | EU254477/NC_010776 | [14] |
| *Fagopyrum esculentum* | Ingroup | | Polygonaceae | *Fagopyrum* | MT364821 | [15] |
| *Persicaria chinensis* | Ingroup/Outgroup | | Polygonaceae | *Persicaria* | NC_050358/MN627221 | - |
| *Afrobrunnichia erecta* | Ingroup/Outgroup | | Polygonaceae | *Afrobrunnichia* | MH286316 | [16] |
| *Limonium sinense* | Outgroup | | Plumbaginaceae | *Limonium* | MN599096 | [17] |
| *Plumbago auriculata* | Outgroup | | Plumbaginaceae | *Plumbago* | NC_041245/MH286308 | [16] |

**Table S3** Plastome gene content and functional classification in *Fagopyrum*

| **Category for gene** | **Group of gene** | **Name of gene** | | | | | | | **Number** |
| --- | --- | --- | --- | --- | --- | --- | --- | --- | --- |
| Photosynthesis-related genes | Photosystem I | *psaA* | *psaB* | *psaC* | *psaI* | *psaJ* |  | | 5 |
|  | Photosystem II | *psbA* | *psbK* | *psbI* | *psbM* | *psbD* | *psbF* | |  |
|  |  | *psbC* | *psbH* | *psbJ* | *psbL* | *psbE* | *psbB* | *psbN* | 13 |
|  | Cytochrome b/f compelx | *psbT* | *psbZ* | *petN* | *petA* | *petL* | *petG* | |  |
|  |  | *petB** | *petD** |  |  |  |  | | 8 |
|  | ATP synthase | *atpA* | *atpF** | *atpH* | *atpI* | *atpE* | *atpB* | | 6 |
|  | Cytochrome c-type synthesis | *ccsA* |  |  |  |  |  | | 1 |
|  | Assembly/stability of photosystem I | *ycf3*** | *ycf4* |  |  |  |  | | 2 |
|  | NADPH dehydrogenase | *ndhB*^, a^* | *ndhH* | *ndhA** | *ndhI* | *ndhG* | *ndhJ* | |  |
|  |  | *ndhE* | *ndhF* | *ndhC* | *ndhK* | *ndhD* |  | | 10+1*2 |
|  | Rubisco | *rbcL* |  |  |  |  |  | | 1 |
| Transcription and translation related genes RNA genes | Transcription Small subunit of ribosome | *rpoA* | *rpoC2* | *rpoC1** | *rpoB* | *rps16** | *rps7 ^a^* | |  |
|  |  | *rps14* | *rps4* | *rps18* | *rps12*^, a^* | *rps11* | *rps8* | |  |
|  |  | *rps3* | *rps19* | *rps15* | *rps2* |  |  | | 14+2*2 |
|  | Large subunit of ribosome | *rpl33* | *rpl20* | *rpl36* | *rpl14* | *rpl16** | *rpl22* | |  |
|  |  | *rpl2*^, a^* | *rpl23 ^a^* | *rpl32* |  |  |  | | 7+2*2 |
|  | Translational initiation factor | *infA* |  |  |  |  |  | | 1 |
|  | Ribosomal RNA | *rrn16 ^a^* | *rrn23 ^a^* | *rrn4.5 ^a^* | *rrn5 ^a^* |  |  | | 4*2 |
|  | Transfer RNA | *trnH-GUG* | *trnK-UUU** | *trnQ-UUG* | *trnS-GCU* | *trnG-UCC*^, a^* | *trnR-UCU* | |  |
|  |  | *trnC-GCA* | *trnD-GUC* | *trnY-GUA* | *trnE-UUC* | *trnT-GGU* | *trnS-UGA* | |  |
|  |  | *trnfM-CAU ^a^* | *trnS-GGA* | *trnT-UGU* | *trnL-UAA** | *trnF-GAA* | *trnV-UAC** | |  |
|  |  | *trnM-CAU* | *trnW-CCA* | *trnP-UGG* | *trnI-CAU ^a^* | *trnL-CAA ^a^* | *trnV-GAC ^a^* | |  |
|  |  | *trnI-GAU*^, a^* | *trnA-UGC*^, a^* | *trnR-ACG ^a^* | *trnN-GUU ^a^* | *trnL-UAG* |  | | 19+9*2 |
| Other genes | RNA processing | *matK* |  |  |  |  |  | | 1 |
|  | Carbon metabolism | *cemA* |  |  |  |  |  | | 1 |
|  | Fatty acid synthesis | *accD* |  |  |  |  |  | | 1 |
|  | Proteolysis | *clpP*** |  |  |  |  |  | | 1 |
|  | Component of TIC complex | *ycf1 ^a^* |  |  |  |  |  | | 1*2 |
|  | Hypothetical proteins | *ycf2 ^a^* |  |  |  |  |  | | 1*2 |
| Total |  |  |  |  |  |  |  | | 131 |

*Gene with one intron. ** Gene with two introns. ^a^ Gene with two copies.

**Table S4** Plastomes characteristics of Polygonaceae species.

| **NO.** | **Species** | **LSC** | **IRb/IRa** | **SSC** | **Gene** | **Intergenic** | **Plastome size** |
| --- | --- | --- | --- | --- | --- | --- | --- |
| 1 | *Calligonum arborescens* | 87629 | 30526 | 13323 | 114205 | 47799 | 162004 |
| 2 | *Calligonum caput-medusae* | 87632 | 30521 | 13345 | 114211 | 47808 | 162019 |
| 3 | *Calligonum caput-medusae2* | 87636 | 30521 | 13365 | 114230 | 47813 | 162043 |
| 4 | *Calligonum junceum1* | 87650 | 30550 | 13286 | 114202 | 47834 | 162036 |
| 5 | *Calligonum junceum4* | 87750 | 30526 | 13322 | 114215 | 47909 | 162124 |
| 6 | *Calligonum junceum3* | 87707 | 30526 | 13322 | 114215 | 47866 | 162081 |
| 7 | *Calligonum aphyllum2* | 86862 | 30526 | 13347 | 114223 | 47038 | 161261 |
| 8 | *Calligonum aphyllum1* | 86853 | 30526 | 13346 | 114222 | 47029 | 161251 |
| 9 | *Calligonum densum* | 86861 | 30526 | 13351 | 114226 | 47038 | 161264 |
| 10 | *Calligonum cordatum* | 86873 | 30526 | 13351 | 114226 | 47050 | 161276 |
| 11 | *Calligonum juochiangense* | 86941 | 30531 | 13352 | 114229 | 47126 | 161355 |
| 12 | *Calligonum korlaense1* | 86952 | 30516 | 13355 | 114219 | 47120 | 161339 |
| 13 | *Calligonum korlaense2* | 86914 | 30525 | 13352 | 114217 | 47099 | 161316 |
| 14 | *Calligonum klementzii* | 86969 | 30526 | 13384 | 114310 | 47095 | 161405 |
| 15 | *Calligonum colubrinum 2* | 86901 | 30531 | 13358 | 114174 | 47147 | 161321 |
| 16 | *Calligonum gobicum* | 86915 | 30552 | 13356 | 114234 | 47141 | 161375 |
| 17 | *Calligonum ebinuricum3* | 86946 | 30530 | 13358 | 114189 | 47175 | 161364 |
| 18 | *Calligonum ebinuricum2* | 86835 | 30547 | 13361 | 114179 | 47111 | 161290 |
| 19 | *Calligonum ebinuricum1* | 86822 | 30541 | 13361 | 114180 | 47085 | 161265 |
| 20 | *Calligonum ebinuricum4* | 86822 | 30541 | 13361 | 114180 | 47085 | 161265 |
| 21 | *Calligonum jeminaicum* | 88160 | 30528 | 13319 | 114204 | 48331 | 162535 |
| 22 | *Calligonum junceum2* | 88107 | 30468 | 13416 | 114179 | 48280 | 162459 |
| 23 | *Rumex wittrockii* | 84750 | 30651 | 12999 | 113671 | 45380 | 159051 |
| **Table S4 (continued)** | | | | | | | |
| **NO.** | **Species** | **LSC** | **IRb/IRa** | **SSC** | **Gene** | **Intergenic** | **Plastome size** |
| 24 | *Rumex crispus1* | 87213 | 30534 | 13011 | 114240 | 47052 | 161292 |
| 25 | *Rumex crispus2* | 84895 | 30597 | 12762 | 113677 | 45174 | 158851 |
| 26 | *Rumex japonicus2* | 84962 | 30631 | 12999 | 113732 | 45491 | 159223 |
| 27 | *Rumex japonicus1* | 85028 | 30629 | 13006 | 113743 | 45549 | 159292 |
| 28 | *Rumex acetosa* | 85838 | 30651 | 13129 | 113830 | 46439 | 160269 |
| 29 | *Rumex hypogaeus* | 85609 | 30348 | 13108 | 113838 | 45575 | 159413 |
| 30 | *Oxyria digyna* | 85750 | 30888 | 13172 | 114365 | 46333 | 160698 |
| 31 | *Oxyria sinensis1* | 85502 | 30885 | 13132 | 114312 | 46092 | 160404 |
| 32 | *Oxyria sinensis2* | 85502 | 30885 | 13132 | 114312 | 46092 | 160404 |
| 33 | *Rheum pumilum* | 86997 | 30970 | 12812 | 114471 | 47278 | 161749 |
| 34 | *Rheum przewalskyi* | 86542 | 31023 | 13152 | 114507 | 47233 | 161740 |
| 35 | *Rheum nobile* | 86291 | 31061 | 12837 | 114494 | 46756 | 161250 |
| 36 | *Rheum acuminatum1* | 86145 | 30996 | 13169 | 114341 | 46965 | 161306 |
| 37 | *Rheum acuminatum2* | 86909 | 30970 | 12806 | 114451 | 47204 | 161655 |
| 38 | *Rheum racemiferum* | 87152 | 30854 | 12822 | 114408 | 47274 | 161682 |
| 39 | *Rheum tanguticum2* | 86474 | 30938 | 13165 | 114391 | 47124 | 161515 |
| 40 | *Rheum tanguticum1* | 86480 | 30953 | 13106 | 114376 | 47116 | 161492 |
| 41 | *Rheum officinale* | 86508 | 30956 | 13114 | 114366 | 47168 | 161534 |
| 42 | *Rheum palmatum* | 86518 | 30956 | 13111 | 114382 | 47159 | 161541 |
| 43 | *Rheum hotaoense* | 86935 | 30979 | 13155 | 114328 | 47720 | 162048 |
| 44 | *Rheum franzenbachii* | 86946 | 30979 | 12784 | 114331 | 47357 | 161688 |
| 45 | *Atraphaxis irtyschensis* | 88878 | 30915 | 13484 | 114445 | 49747 | 164192 |
| 46 | *Fallopia sachalinensis* | 87704 | 31108 | 13565 | 114467 | 49018 | 163485 |
| 47 | *Muehlenbeckia australis* | 88166 | 30916 | 13486 | 114582 | 48902 | 163484 |
| **Table S4 (continued)** | | | | | | | |
| **NO.** | **Species** | **LSC** | **IRb/IRa** | **SSC** | **Gene** | **Intergenic** | **Plastome size** |
| 48 | *Fagopyrum caudatum* | 84347 | 30844 | 13162 | 114113 | 45084 | 159197 |
| 49 | *Fagopyrum qiangcai* | 84531 | 30729 | 13155 | 114082 | 45062 | 159144 |
| 50 | *Fagopyrum leptopodum* var. *grossii* | 84444 | 30840 | 13219 | 114085 | 45258 | 159343 |
| 51 | *Fagopyrum leptopodum* | 83902 | 30839 | 13188 | 114090 | 44678 | 158768 |
| 52 | *Fagopyrum statice* | 84392 | 30841 | 13191 | 114164 | 45101 | 159265 |
| 53 | *Fagopyrum gracilipes* | 84209 | 30848 | 13192 | 114151 | 44946 | 159097 |
| 54 | *Fagopyrum luojishanense* | 84432 | 30870 | 13093 | 114143 | 45122 | 159265 |
| 55 | *Fagopyrum gracilipes* var. *odontopterum* | 84455 | 30841 | 13160 | 114129 | 45168 | 159297 |
| 56 | *Fagopyrum urophyllum* (lianas) | 84520 | 30846 | 13215 | 114169 | 45258 | 159427 |
| 57 | *Fagopyrum sp* | 84449 | 30848 | 13196 | 114173 | 45168 | 159341 |
| 58 | *Fagopyrum urophyllum* | 84425 | 30846 | 13171 | 114109 | 45179 | 159288 |
| 59 | *Fagopyrum megaspartanium* | 85051 | 30793 | 13348 | 114406 | 45579 | 159985 |
| 60 | *Fagopyrum cymosum* | 85038 | 30792 | 13313 | 114399 | 45536 | 159935 |
| 61 | *Fagopyrum dibotrys1* | 84423 | 30817 | 13263 | 114404 | 44916 | 159320 |
| 62 | *Fagopyrum dibotrys2* | 84494 | 30794 | 13243 | 114349 | 44976 | 159325 |
| 63 | *Fagopyrum dibotrys3* | 85135 | 30738 | 13308 | 114321 | 45598 | 159919 |
| 64 | *Fagopyrum tataricum* | 84398 | 30817 | 13240 | 114360 | 44912 | 159272 |
| 65 | *Fagopyrum tataricum* (cultivar Miqiao) | 84398 | 30817 | 13240 | 114360 | 44912 | 159272 |
| 66 | *Fagopyrum esculentum* | 84876 | 30685 | 13330 | 114192 | 45384 | 159576 |
| 67 | *Fagopyrum esculentum* subsp. *ancestrale* | 84885 | 30685 | 13344 | 114199 | 45400 | 159599 |
| 68 | *Persicaria chinensis* | 84347 | 30872 | 12890 | 113935 | 45046 | 158981 |
| 69 | *Afrobrunnichia erecta* | 87317 | 34631 | 13653 | 117858 | 52374 | 170232 |

**Table S5** The rate of synonymous (Ks) and non-synonymous (Ka) substitutions protein-coding genes of the *Fagopyrum* plastomes

| **Gene** | **Species** | **Ks** | **Ka** | **Ka/Ks** |
| --- | --- | --- | --- | --- |
| *psaA* | (109) *F. tataricum* | 0.0833 | 0.0006 | 0.0072 |
|  | (246) *F.* *esculentum* | 0.0859 | 0.0012 | 0.0140 |
|  | (815) *F. cymosum* | 0.0833 | 0.0012 | 0.0144 |
|  | (802) *F. gracilipes* | 0.0075 | 0 | 0 |
|  | (821) *F. leptopodum* | 0.0094 | 0.0006 | 0.0638 |
|  | (837) *F. caudatum* | 0.0075 | 0 | 0 |
| *psaB* | *F. tataricum* | 0.0877 | 0.0018 | 0.0205 |
|  | *F.* *esculentum* | 0.0944 | 0.0030 | 0.0318 |
|  | *F. cymosum* | 0.0855 | 0.0018 | 0.0211 |
|  | *F. gracilipes* | 0.0060 | 0.0006 | 0.1000 |
|  | *F. leptopodum* | 0.0079 | 0.0006 | 0.0759 |
|  | *F. caudatum* | 0.0120 | 0.0006 | 0.0500 |
| *psaC* | *F. tataricum* | 0.1473 | 0 | 0 |
|  | *F.* *esculentum* | 0.1671 | 0 | 0 |
|  | *F. cymosum* | 0.1473 | 0 | 0 |
|  | *F. gracilipes* | 0.0169 | 0 | 0 |
|  | *F. leptopodum* | 0.0169 | 0 | 0 |
|  | *F. caudatum* | 0 | 0 | N/A |
| *psaI* | *F. tataricum* | 0 | 0.0246 | N/A |
|  | *F.* *esculentum* | 0 | 0.0122 | N/A |
|  | *F. cymosum* | 0 | 0.0122 | N/A |
|  | *F. gracilipes* | 0 | 0 | N/A |
|  | *F. leptopodum* | 0 | 0.0123 | N/A |
|  | *F. caudatum* | 0 | 0 | N/A |
| *psaJ* | *F. tataricum* | 0.0888 | 0.0102 | 0.1149 |
|  | *F.* *esculentum* | 0.1895 | 0.0102 | 0.0538 |
|  | *F. cymosum* | 0.0888 | 0.0102 | 0.1149 |
|  | *F. gracilipes* | 0.0284 | 0 | 0 |
|  | *F. leptopodum* | 0.0586 | 0 | 0 |
|  | *F. caudatum* | 0.0287 | 0 | 0 |
| *psbA* | *F. tataricum* | 0.0882 | 0 | 0 |
|  | *F.* *esculentum* | 0.0793 | 0.0012 | 0.0151 |
|  | *F. cymosum* | 0.0927 | 0 | 0 |
|  | *F. gracilipes* | 0.0120 | 0 | 0 |
|  | *F. leptopodum* | 0.0040 | 0 | 0 |
|  | *F. caudatum* | 0 | 0 | N/A |
| *psbB* | *F. tataricum* | 0.1051 | 0.0052 | 0.0495 |
|  | *F.* *esculentum* | 0.0957 | 0.0043 | 0.0449 |
|  | *F. cymosum* | 0.1051 | 0.0052 | 0.0495 |
|  | *F. gracilipes* | 0.0193 | 0.0009 | 0.0466 |
|  | *F. leptopodum* | 0.0137 | 0.0009 | 0.0657 |
|  | *F. caudatum* | 0.0137 | 0.0009 | 0.0657 |
| *psbC* | *F. tataricum* | 0.0735 | 0 | 0 |
|  | *F.* *esculentum* | 0.0798 | 0.0010 | 0.0125 |
|  | *F. cymosum* | 0.0118 | 0 | 0 |
|  | *F. gracilipes* | 0 | 0 | N/A |
|  | *F. leptopodum* | 0.0118 | 0 | 0 |
|  | *F. caudatum* | 0.0029 | 0 | 0 |
| *psbD* | *F. tataricum* | 0.0534 | 0.0025 | 0.0468 |
|  | *F.* *esculentum* | 0.0577 | 0.0025 | 0.0433 |
|  | *F. cymosum* | 0.0534 | 0.0025 | 0.0468 |
|  | *F. gracilipes* | 0 | 0.0012 | N/A |
|  | *F. leptopodum* | 0 | 0 | N/A |
|  | *F. caudatum* | 0.00040 | 0 | 0 |
| *psbE* | *F. tataricum* | 0.0332 | 0 | 0 |
|  | *F.* *esculentum* | 0.0332 | 0 | 0 |
|  | *F. cymosum* | 0.0332 | 0 | 0 |
|  | *F. gracilipes* | 0.0332 | 0 | 0 |
|  | *F. leptopodum* | 0 | 0 | N/A |
|  | *F. caudatum* | 0 | 0 | N/A |
| *psbF* | *F. tataricum* | 0.0949 | 0.0117 | 0.1233 |
|  | *F.* *esculentum* | 0.0949 | 0.0117 | 0.1233 |
|  | *F. cymosum* | 0.0949 | 0.0117 | 0.1233 |
|  | *F. gracilipes* | 0 | 0 | N/A |
|  | *F. leptopodum* | 0 | 0 | N/A |
|  | *F. caudatum* | 0 | 0 | N/A |
| *psbH* | *F. tataricum* | 0.0733 | 0.0247 | 0.3370 |
|  | *F.* *esculentum* | 0.0929 | 0.0310 | 0.3337 |
|  | *F. cymosum* | 0.0543 | 0.0247 | 0.4549 |
|  | *F. gracilipes* | 0 | 0 | N/A |
|  | *F. leptopodum* | 0 | 0 | N/A |
|  | *F. caudatum* | 0.0175 | 0 | 0 |
| *psbI* | *F. tataricum* | 0.0343 | 0 | 0 |
|  | *F.* *esculentum* | 0.0345 | 0 | 0 |
|  | *F. cymosum* | 0.0343 | 0 | 0 |
|  | *F. gracilipes* | 0 | 0 | N/A |
|  | *F. leptopodum* | 0 | 0 | N/A |
|  | *F. caudatum* | 0 | 0 | N/A |
| *psbJ* | *F. tataricum* | 0.0883 | 0 | 0 |
|  | *F.* *esculentum* | 0.0875 | 0.0116 | 0.1326 |
|  | *F. cymosum* | 0.0883 | 0 | 0 |
|  | *F. gracilipes* | 0 | 0 | N/A |
|  | *F. leptopodum* | 0.0284 | 0 | 0 |
|  | *F. caudatum* | 0 | 0 | N/A |
| *psbK* | *F. tataricum* | 0.1486 | 0.0215 | 0.1447 |
|  | *F.* *esculentum* | 0.1486 | 0.0215 | 0.1447 |
|  | *F. cymosum* | 0.1486 | 0.0215 | 0.1447 |
|  | *F. gracilipes* | 0.0467 | 0 | 0 |
|  | *F. leptopodum* | 0.0230 | 0 | 0 |
|  | *F. caudatum* | 0.0230 | 0.0071 | 0.3087 |
| *psbL* | *F. tataricum* | 0 | 0 | N/A |
|  | *F.* *esculentum* | 0 | 0 | N/A |
|  | *F. cymosum* | 0 | 0 | N/A |
|  | *F. gracilipes* | 0 | 0 | N/A |
|  | *F. leptopodum* | 0.0362 | 0 | 0 |
|  | *F. caudatum* | 0 | 0 | N/A |
| *psbM* | *F. tataricum* | 0.0370 | 0 | 0 |
|  | *F.* *esculentum* | 0.0370 | 0 | 0 |
|  | *F. cymosum* | 0.0370 | 0 | 0 |
|  | *F. gracilipes* | 0 | 0 | N/A |
|  | *F. leptopodum* | 0 | 0 | N/A |
|  | *F. caudatum* | 0 | 0 | N/A |
| *psbN* | *F. tataricum* | 0.0290 | 0 | 0 |
|  | *F.* *esculentum* | 0.0290 | 0 | 0 |
|  | *F. cymosum* | 0.0290 | 0 | 0 |
|  | *F. gracilipes* | 0 | 0 | N/A |
|  | *F. leptopodum* | 0 | 0 | N/A |
|  | *F. caudatum* | 0 | 0 | N/A |
| *psbT* | *F. tataricum* | 0.0377 | 0.0272 | 0.7215 |
|  | *F.* *esculentum* | 0 | 0.0273 | N/A |
|  | *F. cymosum* | 0.0775 | 0.0272 | 0.3510 |
|  | *F. gracilipes* | 0 | 0 | N/A |
|  | *F. leptopodum* | 0.0385 | 0 | 0 |
|  | *F. caudatum* | 0 | 0 | N/A |
| *psbZ* | *F. tataricum* | 0.0421 | 0.0144 | 0.3420 |
|  | *F.* *esculentum* | 0.0421 | 0.0144 | 0.3420 |
|  | *F. cymosum* | 0.0421 | 0.0144 | 0.3420 |
|  | *F. gracilipes* | 0 | 0 | N/A |
|  | *F. leptopodum* | 0 | 0 | N/A |
|  | *F. caudatum* | 0 | 0 | N/A |
| *petA* | *F. tataricum* | 0.0918 | 0.0124 | 0.1351 |
|  | *F.* *esculentum* | 0.0870 | 0.0124 | 0.1425 |
|  | *F. cymosum* | 0.0870 | 0.0124 | 0.1425 |
|  | *F. gracilipes* | 0.0087 | 0.0014 | 0.1609 |
|  | *F. leptopodum* | 0.0131 | 0 | 0 |
|  | *F. caudatum* | 0.0175 | 0.0014 | 0.0800 |
| *petB* | *F. tataricum* | 0.0854 | 0 | 0 |
|  | *F.* *esculentum* | 0.0785 | 0 | 0 |
|  | *F. cymosum* | 0.0854 | 0 | 0 |
|  | *F. gracilipes* | 0.0062 | 0 | 0 |
|  | *F. leptopodum* | 0 | 0 | N/A |
|  | *F. caudatum* | 0.0125 | 0 | 0 |
| *petD* | *F. tataricum* | 0.1160 | 0.0028 | 0.0241 |
|  | *F.* *esculentum* | 0.1345 | 0 | 0 |
|  | *F. cymosum* | 0.1151 | 0 | 0 |
|  | *F. gracilipes* | 0.0423 | 0 | 0 |
|  | *F. leptopodum* | 0.0336 | 0 | 0 |
|  | *F. caudatum* | 0 | 0 | N/A |
| *petG* | *F. tataricum* | 0.1030 | 0.0122 | 0.1184 |
|  | *F.* *esculentum* | 0.1406 | 0.0122 | 0.0868 |
|  | *F. cymosum* | 0.1030 | 0.0122 | 0.1184 |
|  | *F. gracilipes* | 0 | 0 | N/A |
|  | *F. leptopodum* | 0 | 0 | N/A |
|  | *F. caudatum* | 0 | 0 | N/A |
| *petL* | *F. tataricum* | 0.1110 | 0.0305 | 0.2748 |
|  | *F.* *esculentum* | 0.0347 | 0.0465 | 1.3401 |
|  | *F. cymosum* | 0.1110 | 0.0305 | 0.2748 |
|  | *F. gracilipes* | 0 | 0 | N/A |
|  | *F. leptopodum* | 0 | 0 | N/A |
|  | *F. caudatum* | 0 | 0 | N/A |
| *petN* | *F. tataricum* | 0.0435 | 0 | 0 |
|  | *F.* *esculentum* | 0.0435 | 0 | 0 |
|  | *F. cymosum* | 0.0435 | 0 | 0 |
|  | *F. gracilipes* | 0 | 0 | N/A |
|  | *F. leptopodum* | 0 | 0 | N/A |
|  | *F. caudatum* | 0 | 0 | N/A |
| *atpA* | *F. tataricum* | 0.0246 | 0.0053 | 0.2154 |
|  | *F.* *esculentum* | 0.1357 | 0.0070 | 0.0516 |
|  | *F. cymosum* | 0.1246 | 0.0088 | 0.0706 |
|  | *F. gracilipes* | 0.0190 | 0.0009 | 0.0474 |
|  | *F. leptopodum* | 0.0273 | 0.0026 | 0.0952 |
|  | *F. caudatum* | 0.0273 | 0.0009 | 0.0330 |
| *atpB* | *F. tataricum* | 0.1022 | 0.0027 | 0.0264 |
|  | *F.* *esculentum* | 0.1085 | 0.0045 | 0.0415 |
|  | *F. cymosum* | 0.1053 | 0.0018 | 0.0171 |
|  | *F. gracilipes* | 0.0134 | 0.0018 | 0.1343 |
|  | *F. leptopodum* | 0.0188 | 0.0009 | 0.0479 |
|  | *F. caudatum* | 0.0080 | 0 | 0 |
| *atpE* | *F. tataricum* | 0.1048 | 0.0218 | 0.2080 |
|  | *F.* *esculentum* | 0.1046 | 0.0219 | 0.2094 |
|  | *F. cymosum* | 0.1049 | 0.0218 | 0.2078 |
|  | *F. gracilipes* | 0.0200 | 0.0066 | 0.3300 |
|  | *F. leptopodum* | 0.0209 | 0.0099 | 0.4737 |
|  | *F. caudatum* | 0.0314 | 0.0066 | 0.2102 |
| *atpF* | *F. tataricum* | 0.0564 | 0.0287 | 0.5089 |
|  | *F.* *esculentum* | 0.0736 | 0.0262 | 0.3560 |
|  | *F. cymosum* | 0.0648 | 0.0263 | 0.4059 |
|  | *F. gracilipes* | 0.0077 | 0.0047 | 0.6104 |
|  | *F. leptopodum* | 0 | 0.0071 | N/A |
|  | *F. caudatum* | 0.0235 | 0.0047 | 0.2000 |
| *atpH* | *F. tataricum* | 0.0608 | 0 | 0 |
|  | *F.* *esculentum* | 0.0768 | 0 | 0 |
|  | *F. cymosum* | 0.0608 | 0 | 0 |
|  | *F. gracilipes* | 0 | 0 | N/A |
|  | *F. leptopodum* | 0 | 0 | N/A |
|  | *F. caudatum* | 0.0148 | 0 | 0 |
| *atpI* | *F. tataricum* | 0.1054 | 0.0054 | 0.0512 |
|  | *F.* *esculentum* | 0.0928 | 0.0072 | 0.0776 |
|  | *F. cymosum* | 0.1052 | 0.0072 | 0.0684 |
|  | *F. gracilipes* | 0.0166 | 0.0018 | 0.1084 |
|  | *F. leptopodum* | 0.0166 | 0.0018 | 0.1084 |
|  | *F. caudatum* | 0.0166 | 0.0018 | 0.1084 |
| *ndhA* | *F. tataricum* | 0.1460 | 0.0199 | 0.1363 |
|  | *F.* *esculentum* | 0.1569 | 0.0234 | 0.1491 |
|  | *F. cymosum* | 0.1457 | 0.0212 | 0.1455 |
|  | *F. gracilipes* | 0.0036 | 0.0012 | 0.3333 |
|  | *F. leptopodum* | 0.0072 | 0.0049 | 0.6806 |
|  | *F. caudatum* | 0.0072 | 0.0025 | 0.3472 |
| *ndhB* | *F. tataricum* | 0.0080 | 0.0026 | 0.3250 |
|  | *F.* *esculentum* | 0.0107 | 0.0035 | 0.3271 |
|  | *F. cymosum* | 0.0080 | 0.0026 | 0.3250 |
|  | *F. gracilipes* | 0 | 0 | N/A |
|  | *F. leptopodum* | 0 | 0 | N/A |
|  | *F. caudatum* | 0 | 0.0009 | N/A |
| *ndhC* | *F. tataricum* | 0.0768 | 0.0129 | 0.1680 |
|  | *F.* *esculentum* | 0.0890 | 0.0166 | 0.1865 |
|  | *F. cymosum* | 0.0770 | 0.0092 | 0.1195 |
|  | *F. gracilipes* | 0.0114 | 0 | 0 |
|  | *F. leptopodum* | 0.0114 | 0 | 0 |
|  | *F. caudatum* | 0.0348 | 0.0073 | 0.2098 |
| *ndhD* | *F. tataricum* | 0.0988 | 0.0200 | 0.2024 |
|  | *F.* *esculentum* | 0.1150 | 0.0210 | 0.1826 |
|  | *F. cymosum* | 0.1019 | 0.0218 | 0.2139 |
|  | *F. gracilipes* | 0.0028 | 0.0035 | 1.2500 |
|  | *F. leptopodum* | 0.0083 | 0.0044 | 0.5301 |
|  | *F. caudatum* | 0.0111 | 0.0044 | 0.3964 |
| *ndhE* | *F. tataricum* | 0.1304 | 0.0175 | 0.1342 |
|  | *F.* *esculentum* | 0.1304 | 0.0087 | 0.0667 |
|  | *F. cymosum* | 0.1304 | 0.0175 | 0.1342 |
|  | *F. gracilipes* | 0 | 0 | N/A |
|  | *F. leptopodum* | 0.0135 | 0 | 0 |
|  | *F. caudatum* | 0.0135 | 0 | 0 |
| *ndhF* | *F. tataricum* | 0.1220 | 0.0423 | 0.3467 |
|  | *F.* *esculentum* | 0.1277 | 0.0469 | 0.3673 |
|  | *F. cymosum* | 0.1218 | 0.0423 | 0.3473 |
|  | *F. gracilipes* | 0.0097 | 0.0058 | 0.5979 |
|  | *F. leptopodum* | 0.0177 | 0.0082 | 0.4633 |
|  | *F. caudatum* | 0.0195 | 0.0064 | 0.3282 |
| *ndhG* | *F. tataricum* | 0.1546 | 0.0202 | 0.1307 |
|  | *F.* *esculentum* | 0.1644 | 0.0279 | 0.1697 |
|  | *F. cymosum* | 0.1546 | 0.0202 | 0.1307 |
|  | *F. gracilipes* | 0 | 0.0050 | N/A |
|  | *F. leptopodum* | 0.0157 | 0.0050 | 0.3185 |
|  | *F. caudatum* | 0.0078 | 0 | 0 |
| *ndhH* | *F. tataricum* | 0.1653 | 0.0216 | 0.1307 |
|  | *F.* *esculentum* | 0.1887 | 0.0194 | 0.1028 |
|  | *F. cymosum* | 0.1843 | 0.0216 | 0.1172 |
|  | *F. gracilipes* | 0.0151 | 0.0044 | 0.2914 |
|  | *F. leptopodum* | 0.0306 | 0.0033 | 0.1078 |
|  | *F. caudatum* | 0.0113 | 0.0022 | 0.1947 |
| *ndhI* | *F. tataricum* | 0.0966 | 0.0103 | 0.1066 |
|  | *F.* *esculentum* | 0.1152 | 0.0103 | 0.0894 |
|  | *F. cymosum* | 0.0966 | 0.0103 | 0.1066 |
|  | *F. gracilipes* | 0.0253 | 0.0026 | 0.1028 |
|  | *F. leptopodum* | 0.0168 | 0.0051 | 0.3036 |
|  | *F. caudatum* | 0.0337 | 0 | 0 |
| *ndhJ* | *F. tataricum* | 0.0552 | 0.0083 | 0.1504 |
|  | *F.* *esculentum* | 0.0457 | 0.0028 | 0.0613 |
|  | *F. cymosum* | 0.0552 | 0.0083 | 0.1504 |
|  | *F. gracilipes* | 0.0089 | 0.0028 | 0.3146 |
|  | *F. leptopodum* | 0.0089 | 0 | 0 |
|  | *F. caudatum* | 0.0089 | 0 | 0 |
| *ndhK* | *F. tataricum* | 0.0952 | 0.0253 | 0.2658 |
|  | *F.* *esculentum* | 0.0913 | 0.0242 | 0.2651 |
|  | *F. cymosum* | 0.0952 | 0.0253 | 0.2658 |
|  | *F. gracilipes* | 0 | 0 | N/A |
|  | *F. leptopodum* | 0.0056 | 0.0018 | 0.3214 |
|  | *F. caudatum* | 0.0056 | 0.0035 | 0.6250 |
| *rps2* | *F. tataricum* | 0.0953 | 0.0149 | 0.1563 |
|  | *F.* *esculentum* | 0.0891 | 0.0149 | 0.1672 |
|  | *F. cymosum* | 0.0885 | 0.0149 | 0.1684 |
|  | *F. gracilipes* | 0.0061 | 0 | 0 |
|  | *F. leptopodum* | 0.0185 | 0.0018 | 0.0973 |
|  | *F. caudatum* | 0.0184 | 0 | 0 |
| *rps3* | *F. tataricum* | 0.1224 | 0.0139 | 0.1136 |
|  | *F.* *esculentum* | 0.1186 | 0.0170 | 0.1433 |
|  | *F. cymosum* | 0.1034 | 0.0170 | 0.1644 |
|  | *F. gracilipes* | 0.0067 | 0.0040 | 0.5970 |
|  | *F. leptopodum* | 0.0135 | 0.0040 | 0.2963 |
|  | *F. caudatum* | 0 | 0.0020 | N/A |
| *rps4* | *F. tataricum* | 0.0650 | 0.0067 | 0.1031 |
|  | *F.* *esculentum* | 0.0800 | 0.0111 | 0.1388 |
|  | *F. cymosum* | 0.0650 | 0.0067 | 0.1031 |
|  | *F. gracilipes* | 0 | 0.0022 | N/A |
|  | *F. leptopodum* | 0 | 0 | N/A |
|  | *F. caudatum* | 0.0139 | 0 | 0 |
| *rps7* | *F. tataricum* | 0.0350 | 0 | 0 |
|  | *F.* *esculentum* | 0.0173 | 0 | 0 |
|  | *F. cymosum* | 0.0350 | 0 | 0 |
|  | *F. gracilipes* | 0.0086 | 0 | 0 |
|  | *F. leptopodum* | 0.0086 | 0 | 0 |
|  | *F. caudatum* | 0 | 0 | N/A |
| *rps8* | *F. tataricum* | 0.0987 | 0.0184 | 0.1864 |
|  | *F.* *esculentum* | 0.1101 | 0.0286 | 0.2598 |
|  | *F. cymosum* | 0.0987 | 0.0184 | 0.1864 |
|  | *F. gracilipes* | 0 | 0 | N/A |
|  | *F. leptopodum* | 0 | 0.0033 | N/A |
|  | *F. caudatum* | 0.0198 | 0.0066 | 0.3333 |
| *rps11* | *F. tataricum* | 0.1854 | 0.0065 | 0.0351 |
|  | *F.* *esculentum* | 0.1854 | 0.0098 | 0.0529 |
|  | *F. cymosum* | 0.1854 | 0.0065 | 0.0351 |
|  | *F. gracilipes* | 0.0185 | 0 | 0 |
|  | *F. leptopodum* | 0.0373 | 0 | 0 |
|  | *F. caudatum* | 0.0374 | 0 | 0 |
| *rps12* | *F. tataricum* | 0.0304 | 0.0037 | 0.1217 |
|  | *F.* *esculentum* | 0.1010 | 0.0990 | 0.9802 |
|  | *F. cymosum* | 0.0304 | 0 | 0 |
|  | *F. gracilipes* | 0.0100 | 0 | 0 |
|  | *F. leptopodum* | 0.0691 | 0.0991 | 1.4342 |
|  | *F. caudatum* | 0.0590 | 0.0931 | 1.5780 |
| *rps14* | *F. tataricum* | 0.0604 | 0.0261 | 0.4321 |
|  | *F.* *esculentum* | 0.0599 | 0.0217 | 0.3623 |
|  | *F. cymosum* | 0.0599 | 0.0217 | 0.3623 |
|  | *F. gracilipes* | 0 | 0 | N/A |
|  | *F. leptopodum* | 0 | 0 | N/A |
|  | *F. caudatum* | 0.0145 | 0 | 0 |
| *rps15* | *F. tataricum* | 0.2446 | 0.0430 | 0.1758 |
|  | *F.* *esculentum* | 0.02691 | 0.0430 | 1.5979 |
|  | *F. cymosum* | 0.2700 | 0.0429 | 0.1589 |
|  | *F. gracilipes* | 0.0173 | 0.0051 | 0.2948 |
|  | *F. leptopodum* | 0.0173 | 0 | 0 |
|  | *F. caudatum* | 0.0173 | 0 | 0 |
| *rps16* | *F. tataricum* | 0.0481 | 0.0235 | 0.4886 |
|  | *F.* *esculentum* | 0.0483 | 0.0355 | 0.7350 |
|  | *F. cymosum* | 0.0485 | 0.0294 | 0.6062 |
|  | *F. gracilipes* | 0 | 0 | N/A |
|  | *F. leptopodum* | 0 | 0 | N/A |
|  | *F. caudatum* | 0 | 0.0058 | N/A |
| *rps18* | *F. tataricum* | 0.0414 | 0.0043 | 0.1039 |
|  | *F.* *esculentum* | 0.0706 | 0.0043 | 0.0609 |
|  | *F. cymosum* | 0.0273 | 0.0043 | 0.1575 |
|  | *F. gracilipes* | 0 | 0.0043 | N/A |
|  | *F. leptopodum* | 0 | 0 | N/A |
|  | *F. caudatum* | 0.0135 | 0 | 0 |
| *rps19* | *F. tataricum* | 0.1462 | 0.0143 | 0.0978 |
|  | *F.* *esculentum* | 0.1294 | 0.0143 | 0.1105 |
|  | *F. cymosum* | 0.1285 | 0.0143 | 0.1113 |
|  | *F. gracilipes* | 0.0304 | 0 | 0 |
|  | *F. leptopodum* | 0.0304 | 0 | 0 |
|  | *F. caudatum* | 0.0150 | 0 | 0 |
| *rpoA* | *F. tataricum* | 0.1045 | 0.0325 | 0.3110 |
|  | *F.* *esculentum* | 0.0919 | 0.0319 | 0.3471 |
|  | *F. cymosum* | 0.1044 | 0.0325 | 0.3113 |
|  | *F. gracilipes* | 0.0044 | 0.0064 | 1.4545 |
|  | *F. leptopodum* | 0.0135 | 0.0051 | 0.3778 |
|  | *F. caudatum* | 0.0135 | 0.0025 | 0.1852 |
| *rpoB* | *F. tataricum* | 0.0757 | 0.0158 | 0.2087 |
|  | *F.* *esculentum* | 0.0734 | 0.0161 | 0.2193 |
|  | *F. cymosum* | 0.0742 | 0.0163 | 0.2197 |
|  | *F. gracilipes* | 0.0134 | 0.0024 | 0.1791 |
|  | *F. leptopodum* | 0.0107 | 0.0033 | 0.3084 |
|  | *F. caudatum* | 0.0093 | 0.0024 | 0.2581 |
| *rpoC1* | *F. tataricum* | 0.0918 | 0.0165 | 0.1797 |
|  | *F.* *esculentum* | 0.0721 | 0.0136 | 0.1886 |
|  | *F. cymosum* | 0.0872 | 0.0159 | 0.1823 |
|  | *F. gracilipes* | 0.0147 | 0.0019 | 0.1293 |
|  | *F. leptopodum* | 0.0168 | 0.0019 | 0.1131 |
|  | *F. caudatum* | 0.0168 | 0.0019 | 0.1131 |
| *rpoC2* | *F. tataricum* | 0.0987 | 0.0314 | 0.3181 |
|  | *F.* *esculentum* | 0.1028 | 0.0334 | 0.3249 |
|  | *F. cymosum* | 0.0989 | 0.0315 | 0.3185 |
|  | *F. gracilipes* | 0.0042 | 0.0048 | 1.1429 |
|  | *F. leptopodum* | 0.0104 | 0.0039 | 0.3750 |
|  | *F. caudatum* | 0.0105 | 0.0043 | 0.4095 |
| *ycf1* | *F. tataricum* | 0.0206 | 0.0164 | 0.7961 |
|  | *F.* *esculentum* | 0.0206 | 0.0176 | 0.8544 |
|  | *F. cymosum* | 0.0198 | 0.0165 | 0.8333 |
|  | *F. gracilipes* | 0.0025 | 0.0025 | 1.0000 |
|  | *F. leptopodum* | 0.0033 | 0.0023 | 0.6970 |
|  | *F. caudatum* | 0.0048 | 0.0055 | 1.1458 |
| *ycf2* | *F. tataricum* | 0.0127 | 0.0149 | 1.1732 |
|  | *F.* *esculentum* | 0.0134 | 0.0142 | 1.0597 |
|  | *F. cymosum* | 0.0127 | 0.0149 | 1.1732 |
|  | *F. gracilipes* | 0.0043 | 0.0010 | 0.2326 |
|  | *F. leptopodum* | 0.0036 | 0.0016 | 0.4444 |
|  | *F. caudatum* | 0.0014 | 0.0027 | 1.9286 |
| *ycf3* | *F. tataricum* | 0.0171 | 0.0239 | 1.3977 |
|  | *F.* *esculentum* | 0.0984 | 0 | 0 |
|  | *F. cymosum* | 0.0887 | 0.0026 | 0.0293 |
|  | *F. gracilipes* | 0.0084 | 0 | 0 |
|  | *F. leptopodum* | 0.0084 | 0 | 0 |
|  | *F. caudatum* | 0 | 0 | N/A |
| *ycf4* | *F. tataricum* | 0.0633 | 0.0095 | 0.1501 |
|  | *F.* *esculentum* | 0.0635 | 0.0143 | 0.2252 |
|  | *F. cymosum* | 0.0715 | 0.0095 | 0.1329 |
|  | *F. gracilipes* | 0.0231 | 0.0024 | 0.1039 |
|  | *F. leptopodum* | 0.0231 | 0.0024 | 0.1039 |
|  | *F. caudatum* | 0.0153 | 0.0024 | 0.1569 |
| *rpl2* | *F. tataricum* | 0.0098 | 0.0032 | 0.3265 |
|  | *F.* *esculentum* | 0.0049 | 0.0032 | 0.6531 |
|  | *F. cymosum* | 0.0098 | 0.0016 | 0.1633 |
|  | *F. gracilipes* | 0 | 0 | N/A |
|  | *F. leptopodum* | 0 | 0.0016 | N/A |
|  | *F. caudatum* | 0.0049 | 0 | 0 |
| *rpl14* | *F. tataricum* | 0.0809 | 0.0109 | 0.1347 |
|  | *F.* *esculentum* | 0.0809 | 0.0109 | 0.1347 |
|  | *F. cymosum* | 0.0688 | 0.0109 | 0.1584 |
|  | *F. gracilipes* | 0 | 0 | N/A |
|  | *F. leptopodum* | 0.0110 | 0 | 0 |
|  | *F. caudatum* | 0.0110 | 0 | 0 |
| *rpl16* | *F. tataricum* | 0.1545 | 0.0033 | 0.0214 |
|  | *F.* *esculentum* | 0.1210 | 0.0033 | 0.0273 |
|  | *F. cymosum* | 0.1545 | 0.0033 | 0.0214 |
|  | *F. gracilipes* | 0.0093 | 0 | 0 |
|  | *F. leptopodum* | 0.0093 | 0.0033 | 0.3548 |
|  | *F. caudatum* | 0.0282 | 0 | 0 |
| *rpl20* | *F. tataricum* | 0.0686 | 0.0339 | 0.4942 |
|  | *F.* *esculentum* | 0.0929 | 0.0305 | 0.3283 |
|  | *F. cymosum* | 0.0808 | 0.0339 | 0.4196 |
|  | *F. gracilipes* | 0 | 0.0067 | N/A |
|  | *F. leptopodum* | 0.0110 | 0.0100 | 0.9091 |
|  | *F. caudatum* | 0.0223 | 0.0134 | 0.6009 |
| *rpl22* | *F. tataricum* | 0.0829 | 0.0251 | 0.3028 |
|  | *F.* *esculentum* | 0.1153 | 0.0161 | 0.1396 |
|  | *F. cymosum* | 0.0933 | 0.0251 | 0.2690 |
|  | *F. gracilipes* | 0.0190 | 0.0029 | 0.1526 |
|  | *F. leptopodum* | 0 | 0 | N/A |
|  | *F. caudatum* | 0.0286 | 0.0058 | 0.2028 |
| *rpl23* | *F. tataricum* | 0 | 0.0101 | N/A |
|  | *F.* *esculentum* | 0 | 0.0101 | N/A |
|  | *F. cymosum* | 0 | 0.0101 | N/A |
|  | *F. gracilipes* | 0 | 0 | N/A |
|  | *F. leptopodum* | 0 | 0 | N/A |
|  | *F. caudatum* | 0 | 0 | N/A |
| *rpl32* | *F. tataricum* | 0.0803 | 0.0078 | 0.0971 |
|  | *F.* *esculentum* | 0.1066 | 0.0305 | 0.2861 |
|  | *F. cymosum* | 0.0803 | 0.0078 | 0.0971 |
|  | *F. gracilipes* | 0 | 0 | N/A |
|  | *F. leptopodum* | 0 | 0.0076 | N/A |
|  | *F. caudatum* | 0.0248 | 0.0152 | 0.6129 |
| *rpl33* | *F. tataricum* | 0.0880 | 0 | 0 |
|  | *F.* *esculentum* | 0.0880 | 0.0066 | 0.0750 |
|  | *F. cymosum* | 0.0880 | 0 | 0 |
|  | *F. gracilipes* | 0 | 0 | N/A |
|  | *F. leptopodum* | 0 | 0 | N/A |
|  | *F. caudatum* | 0 | 0 | N/A |
| *rpl36* | *F. tataricum* | 0 | 0 | N/A |
|  | *F.* *esculentum* | 0 | 0 | N/A |
|  | *F. cymosum* | 0 | 0 | N/A |
|  | *F. gracilipes* | 0 | 0 | N/A |
|  | *F. leptopodum* | 0 | 0 | N/A |
|  | *F. caudatum* | 0 | 0 | N/A |
| *accD* | *F. tataricum* | 0.0930 | 0.0489 | 0.5258 |
|  | *F.* *esculentum* | 0.1048 | 0.0518 | 0.4943 |
|  | *F. cymosum* | 0.0969 | 0.0489 | 0.5046 |
|  | *F. gracilipes* | 0.0169 | 0.0091 | 0.5385 |
|  | *F. leptopodum* | 0.0067 | 0.0091 | 1.3582 |
|  | *F. caudatum* | 0.0068 | 0.0128 | 1.8824 |
| *cemA* | *F. tataricum* | 0.1107 | 0.0403 | 0.3640 |
|  | *F.* *esculentum* | 0.1030 | 0.0444 | 0.4311 |
|  | *F. cymosum* | 0.1106 | 0.0404 | 0.3653 |
|  | *F. gracilipes* | 0.0064 | 0.0019 | 0.2969 |
|  | *F. leptopodum* | 0.0128 | 0.0057 | 0.4453 |
|  | *F. caudatum* | 0.0066 | 0.0019 | 0.2879 |
| *ccsA* | *F. tataricum* | 0.1148 | 0.0336 | 0.2927 |
|  | *F.* *esculentum* | 0.1206 | 0.0350 | 0.2902 |
|  | *F. cymosum* | 0.1147 | 0.0350 | 0.3051 |
|  | *F. gracilipes* | 0.0047 | 0.0040 | 0.8511 |
|  | *F. leptopodum* | 0.0095 | 0.0067 | 0.7053 |
|  | *F. caudatum* | 0.0047 | 0.0080 | 1.7021 |
| *clpP* | *F. tataricum* | 0.0341 | 0.0211 | 0.6188 |
|  | *F.* *esculentum* | 0.0411 | 0.0233 | 0.5669 |
|  | *F. cymosum* | 0.0341 | 0.0211 | 0.6188 |
|  | *F. gracilipes* | 0.0270 | 0.0021 | 0.0778 |
|  | *F. leptopodum* | 0.0134 | 0.0063 | 0.4701 |
|  | *F. caudatum* | 0 | 0.0042 | N/A |
| *infA* | *F. tataricum* | 0.1106 | 0.0173 | 0.1564 |
|  | *F.* *esculentum* | 0.0910 | 0.0173 | 0.1901 |
|  | *F. cymosum* | 0.1106 | 0.0173 | 0.1564 |
|  | *F. gracilipes* | 0.0351 | 0 | 0 |
|  | *F. leptopodum* | 0.0351 | 0 | 0 |
|  | *F. caudatum* | 0.0531 | 0.0057 | 0.1073 |
| *matK* | *F. tataricum* | 0.1203 | 0.0559 | 0.4647 |
|  | *F.* *esculentum* | 0.1224 | 0.0637 | 0.5204 |
|  | *F. cymosum* | 0.1206 | 0.0586 | 0.4859 |
|  | *F. gracilipes* | 0.0085 | 0.0103 | 1.2118 |
|  | *F. leptopodum* | 0.0142 | 0.0094 | 0.6620 |
|  | *F. caudatum* | 0.0200 | 0.0120 | 0.6000 |
| *rbcL* | *F. tataricum* | 0.0694 | 0.0009 | 0.0130 |
|  | *F.* *esculentum* | 0.0887 | 0 | 0 |
|  | *F. cymosum* | 0.0695 | 0 | 0 |
|  | *F. gracilipes* | 0.0087 | 0.0019 | 0.2184 |
|  | *F. leptopodum* | 0.0116 | 0.0019 | 0.1638 |
|  | *F. caudatum* | 0.0116 | 0.0019 | 0.1638 |

**Table S6** Hotspots (gene/intergenic regions) among *Fagopyrum* plastomes.

| **Type** | **gene/IGS** | **length** | **SNPs** | **Indels** | **Pi** |
| --- | --- | --- | --- | --- | --- |
| gene  regions | *rps15* | 295 | 33 | 3 | 0.05215 |
|  | *trnK* | 1152 | 95 | 10 | 0.04237 |
|  | *trnL* | 618 | 54 | 12 | 0.0416 |
|  | *matK* | 1527 | 158 | 1 | 0.0406 |
|  | *ndhA* | 2231 | 174 | 10 | 0.0387 |
|  | *clpP2* | 2309 | 207 | 18 | 0.03801 |
|  | *rpoC2-*2028 | 700 | 60 | 1 | 0.03639 |
|  | *rpl16* | 1494 | 115 | 7 | 0.03625 |
|  | *ndhF* | 2263 | 210 | 6 | 0.03620 |
|  | *petB-*intron | 783 | 70 | 7 | 0.03295 |
|  | *accD* | 1437 | 111 | 5 | 0.03236 |
|  | *ndhH* | 1182 | 90 | 0 | 0.03184 |
|  | *cemA* | 694 | 53 | 2 | 0.03152 |
|  | *trnG* | 804 | 58 | 9 | 0.03133 |
|  | *rps16* | 1197 | 97 | 17 | 0.03113 |
|  | *ndhG* | 531 | 41 | 0 | 0.03079 |
|  | *ccsA* | 966 | 75 | 0 | 0.03030 |
|  | *petD*-intron | 749 | 47 | 5 | 0.02833 |
|  | *rpoC1-*intron | 770 | 49 | 8 | 0.02762 |
|  | *atpF* | 1317 | 87 | 4 | 0.02617 |
| intergenic regions | *rpl32-trnL* | 962 | 154 | 14 | 0.13248 |
|  | *ndhF_rpl32* | 1092 | 160 | 21 | 0.07769 |
|  | *trnE-trnT* | 715 | 86 | 12 | 0.07582 |
|  | *trnD-trnY* | 489 | 80 | 7 | 0.07415 |
|  | *trnP-psaJ* | 529 | 55 | 4 | 0.06953 |
|  | *trnH-psbA* | 420 | 57 | 10 | 0.06531 |
|  | *ndhC-trnV* | 1129 | 112 | 15 | 0.06524 |
|  | *psbM-trnD* | 1211 | 143 | 12 | 0.06206 |
|  | *trnS-trnG* | 1283 | 86 | 13 | 0.06088 |
|  | *ycf3-trnS* | 906 | 108 | 13 | 0.06077 |
|  | *atpH-atpI* | 696 | 85 | 11 | 0.05968 |
|  | *psaJ-rpl33* | 554 | 64 | 7 | 0.05848 |
|  | *rps4-trnT* | 719 | 67 | 10 | 0.05836 |
|  | *trnC-petN* | 1009 | 118 | 9 | 0.05634 |
|  | *trnS-rps4* | 386 | 40 | 4 | 0.05558 |
|  | *accD-psaI* | 750 | 73 | 8 | 0.05555 |
|  | *ndhJ-trnF* | 728 | 68 | 9 | 0.05294 |
|  | *rpoB-trnC* | 1454 | 141 | 18 | 0.05188 |
|  | *trnT-psbD* | 1384 | 148 | 21 | 0.05165 |
|  | *psbE-petL* | 1008 | 101 | 16 | 0.05079 |

**Reference**

1. Song F, Li T, Burgess KS, Feng Y, Ge XJ. Complete plastome sequencing resolves taxonomic relationships among species of *Calligonum* L. (Polygonaceae) in China. BMC Plant Biol. 2020;20:261.
2. Zhou T, Zhu H, Wang J, Xu Y, Wang X. Complete chloroplast genome sequence determination of rheum species and comparative chloroplast genomics for the members of Rumiceae. Plant Cell Rep. 2020;39:811–824.
3. Youn JS, Yang JY, Kim SC, Pak JH. Complete plastome sequence of *Rumex japonicus* (Polygonaceae) in Dok-do Island, Korea. Mitochondrial DNA B. 2019;4:2892–2893.
4. Gui L, Jiang S, Wang H, Nong D, Liu Y. Characterization of the complete chloroplast genome of sorrel (*Rumex acetosa*). Mitochondrial DNA B. 2018;3:904–906.
5. Choi KS, Lee WH, Park SJ. The complete chloroplast genome of Emex australis (Polygonaceae). Mitochondrial DNA B. 2020;5:1431–1432.
6. Zhao KH, Li LQ, Lu YZ, Yang JB, Zhang ZR, Zhao FY, Quan H, Ma XJ, Liao ZH, Lan X. Characterization and Comparative Analysis of Two *Rheum* Complete Chloroplast Genomes. BioMed Res Int. 2020;4:1–11.
7. Fan K, Sun XJ, Huang M, Wang XM. The complete chloroplast genome sequence of the medicinal plant *Rheum palmatum* L. (Polygonaceae). Mitochondrial DNA A. 2016;27:2935–2936.
8. Raman G, Park KT, Nam GH, Kwak M, Park SJ. Characterization of the complete chloroplast genome sequence of the giant knotweed, *Fallopia sachalinensis* from the volcanic island Dokdo, republic of Korea. Mitochondrial DNA Part B. 2019;4:2972–2973.
9. Schuster TM, Gibbs MD, Bayly MJ. Annotated plastome of the temperate woody vine *Muehlenbeckia australis* (G.Forst.) Meisn. (Polygonaceae). Mitochondrial DNA B. 2018;3:399–400.
10. Wang CL, Ding MQ, Zou CY, Zhu XM, Tang Y, Zhou ML, Shao JR. Comparative Analysis of Four Buckwheat Species Based on Morphology and Complete Chloroplast Genome Sequences. Sci. Rep. 2017;7:1–14.
11. Zhang Y, Chen C. The complete chloroplast genome sequence of the medicinal plant *Fagopyrum dibotrys* (Polygonaceae). Mitochondrial DNA B. 2018;3:1087–1089.
12. Cho KS, Yun BK, Yoon YH, Hong SY, Mekapogu M, Kim KH, Yang TJ. Complete chloroplast genome sequence of tartary buckwheat (*Fagopyrum tataricum*) and comparative analysis with common buckwheat (*F. esculentum*). PLoS ONE. 2015;10:1–14.
13. Liu M, Zheng T, Ma Z, Wang D, Wang T, Sun R, He Z, Peng J, Chen H. The complete chloroplast genome sequence of Tartary Buckwheat cultivar Miqiao 1(*Fagopyrum tataricum* Gaertn.). Mitochondrial DNA B. 2016;1:577–578.
14. Logacheva MD, Samigullin TH, Dhingra A, Penin AA. Comparative chloroplast genomics and phylogenetics of *Fagopyrum esculentum* ssp. *ancestrale* - A wild ancestor of cultivated buckwheat. BMC Plant Biol. 2008;8:1–15.
15. Logacheva MD, Schelkunov MI, Fesenko AN, Kasianov AS, Penin AA. Mitochondrial genome of *Fagopyrum esculentum* and the genetic diversity of extranuclear genomes in buckwheat. Plants. 2020;9:618.
16. Yao G, Jin, JJ, Li HT, Yang JB, Mandala VS, Croley M, Mostow R, Douglas NA, Chase MW, Christenhusz MJM, Soltis DE, Soltis PS, Smith SA, Brockington SF, Moore MJ, Yi TS, Li DZ. Plastid phylogenomic insights into the evolution of Caryophyllales. Mol. Phylogenet. Evol. 2019;134:74–86.
17. Li J, Xu B, Yang Q, Wang T, Zhu Q, Lin Y, Liu ZL. The complete chloroplast genome sequence of *Limonium sinense* (Plumbaginaceae). Mitochondrial DNA B. 2020;5:556–557.
